# Using monitoring and mechanistic modeling to improve understanding of eutrophication in a shallow New England estuary

**DOI:** 10.1016/j.jenvman.2024.120478

**Published:** 2024-03-02

**Authors:** Finnian S. Cashel, Christopher D. Knightes, Chris Lupo, Traci Iott, Kelly Streich, Corey J. Conville, Timothy W. Bridges, Ian Dombroski

**Affiliations:** a Oak Ridge Institute for Science and Education Fellow, United States Environmental Protection Agency, Office of Research and Development, Center for Environmental Measurement & Modeling, Atlantic Coastal Environmental Sciences Division, 27 Tarzwell Drive, Narragansett, RI, 02882, USA; b United States Environmental Protection Agency, Office of Research and Development, Center for Environmental Measurement & Modeling, Atlantic Coastal Environmental Sciences Division, 27 Tarzwell Drive, Narragansett, RI, 02882, USA; c RESPEC, 3825 Jet Dr, Rapid City, SD, 57703, USA; d Connecticut Department of Energy and Environmental Protection, Bureau of Water Protection and Land Reuse, 79 Elm Street, Hartford, CT, 06106, USA; e United States Environmental Protection Agency, Region 1, Laboratory Sciences and Applied Science Divisions, 11 Technology Drive, North Chelmsford, MA, 01863, USA; f United States Environmental Protection Agency, Region 1, Water Division, 5 Post Office Square, Suite 100, Boston, MA, 02109, USA

**Keywords:** Numerical modeling, Eutrophication, Hypoxia, Macroalgae, Stratification

## Abstract

Anthropogenic nutrient loading has resulted in eutrophication and habitat degradation within estuaries. Study of eutrophication in estuaries has often focused on larger systems, while there has been increasing interest in understanding the governing processes in smaller systems. In this study, we incorporate both monitoring data and mechanistic modeling to improve our understanding of eutrophication in a small, shallow New England estuary. High-frequency continuous and discrete water quality samples were collected from 2018 to 2020 along a salinity gradient and at varying depth to provide temporal and spatial resolution of the system. Conditions of this estuary were simulated using the Hydrological Simulation Program – FORTRAN (HSPF) and the Water Quality Analysis Simulation Program (WASP) to develop a mechanistic, numerical fate and transport model. Our findings suggest complex hydrodynamics with three distinct salinity gradients and variability in salinity concentration upstream. Simulated and observed nutrient trends demonstrated decreasing total nitrogen concentration moving downstream and low total phosphorus concentration throughout the system. Simulated nutrient depletion and shading via macroalgae suggest their importance in similar modeling initiatives. Dynamic spatiotemporal variability in dissolved oxygen concentrations ([DO]) resulted from hydrodynamic and ecological processes such as large, rapid swings in phytoplankton. Carbonaceous biological oxygen demand was suggested to be the driver of hypoxia in surface waters, while sediment oxygen demand may drive low [DO] in the stratified, benthic waters. These findings suggest that the coordination of monitoring and modeling was important to understanding the governing mechanisms of eutrophication and hypoxia. Insights from this study could be used to support regional management strategies to increase [DO], improve water clarity, and recover indigenous seagrass beds. This work has the potential to inform future study and management of small, complex estuaries.

## 1. Introduction

Estuaries are important aquatic ecosystems due to the ecoservices they provide. Primary production, water filtration, flood and storm protection, and biodiversity are critical ecoservices of healthy coastal environments (Boerema and Meire, 2017; NOAA, 2017). Increases in anthropogenic nutrient loading have resulted in enhanced eutrophication and exacerbated ecosystem degradation (Malone and Newton, 2020; Rabalais et al., 2009). Eutrophication is the excessive input of organic matter into an ecosystem, which can result in increased algae and phytoplankton growth (Nixon, 1995; Bricker et al., 1999). This process has the potential to cause harmful algal blooms, loss of seagrass beds via shading and interspecific competition, coastal acidification, and hypoxia (Bricker et al., 1999; Cardoso et al., 2004). Hypoxia and dead zones have been further observed to decrease the biodiversity and abundance of benthic communities such as sessile invertebrates (Hale et al., 2016). The frequency and intensity of hypoxic events due to eutrophication has increased exponentially in recent decades (Diaz and Rosenberg, 2008). Assessment of systems such as the Adriatic Sea (Djakovac et al., 2015), the Baltic Sea (Conley et al., 2009), and the Chesapeake Bay (Hagy et al., 2004) have documented the negative effects of eutrophication on water quality.

Due to the growing area and frequency of eutrophic events, large efforts have been taken to gain a greater understanding of the mechanisms governing this phenomenon. Both water quality monitoring and modeling have proven to be effective tools in ecological studies. Monitoring studies such as in the Newark Bay Estuary, United States of America (USA) (Jung et al., 2021) collected discrete data on a variety of parameters to empirically assess eutrophication impacts on water quality. Jung et al. found that elevated nutrient concentrations were likely due to sewage effluent, and that hypoxia was present in the Hackensack River. Study of Escambia Bay, USA using high-frequency, continuous water quality sampling found small-scale spatiotemporal variation in biophysical processes affecting diel-cycling of dissolved oxygen such as freshwater inflow, tides, and irradiance (Duvall et al., 2022).

Alongside monitoring studies, mechanistic models are useful tools to study system wide water quality dynamics due to their ability to link stressor and response variables, track changes over time, and fill spatial and temporal gaps in observed data (Wool et al., 2020). Modeling efforts in the Chesapeake Bay, USA (Bever et al., 2021), Ganwol Estuary, South Korea (Kim et al., 2023), Dunajec River, Poland (Żelazny et al., 2023), Gulf of Mexico, USA (Jarvis et al., 2021), and other ecosystems have documented the effectiveness of mechanistic models to understand and simulate water quality. Such models frequently assist in development of regional management strategies that are designed to reduce anthropogenic loadings to encourage ecosystem recovery via improved water quality (Borusk et al., 2002; Żelazny et al., 2023). Additional water quality modeling studies highlight the importance of specific biological processes. Modeling of Greenwich Bay, USA emphasized the influence of macroalgae due to the large impact on dissolved oxygen (DO) production and consumption, nutrient cycling, and other regulatory processes of water quality (Brush and Nixon, 2010). Development of a CE-QUAL-W2 model in the Lower Minnesota River, USA successfully incorporated a sediment diagenesis module to capture the flux of organic matter to the sediments, the mineralization of that matter, and the reactions of those products (Zhang et al., 2015). Despite this, many notable modeling initiatives have not incorporated the impact of macroalgae and sediment diagenesis which generates potential for error regarding processes that contribute to water quality (Wool et al., 2003; Jarvis et al., 2021; Bever et al., 2021).

Most mechanistic modeling initiatives were developed for large estuaries, such as the Chesapeake Bay, as they hold critical value for human development, recreation, and food sources (Edgar et al., 2000). However, the northeast USA alone has hundreds of small embayments and estuaries which would benefit from increased understanding and management via mechanistic modeling. The Pawcatuck River Estuary (PRE) is a small, shallow estuary in the northeastern USA that has been identified as one of the most intensively nitrogen loaded estuaries on the North Atlantic coast (Fulweiler and Nixon, 2005). This system is listed on both the Rhode Island and Connecticut 303(d) list of impaired waters (RIDEM, 2022; Connecticut Department of Energy and Environmental Protection, 2022). Qualitative observations have identified 100% surface coverage of invasive *Cladophora* spp. (macroalgae) in downstream portions of the PRE (Connecticut Department of Energy and Environmental Protection, 2020b). Correspondingly, historical beds of *Zostera marina* (common eelgrass) were overtaken by filamentous macroalgae in the early 1990s (Rollinson et al., 2021).

In this study, we employed a combined monitoring and modeling strategy to assess eutrophication and the temporal and spatial variations of water quality. The monitoring component of this study included collecting discrete and continuous data in along the length of the Pawcatuck River and Little Narragansett Bay (LNB), including samples near the surface and near the bottom. We also constructed a multimedia modeling framework using the Hydrological Simulation Program – FORTRAN (HSPF) for watershed hydrology and loads, and the Water Quality Analysis Simulation Program (WASP) for estuary hydrodynamics and water quality. The aim of this work is to achieve a system-level understanding of the processes governing eutrophication. Here, we (1) develop and investigate the feasibility of the 1-Dimensional (1D) framework and Dynamic Wave to simulate the hydrodynamics of a shallow, tidal dominated estuary, (2) mechanistically infer how incorporating macroalgae as a state variable affects light penetration (water clarity), nutrient and light availability, and phytoplankton within LNB, and (3) analyze observed and simulated [DO], phytoplankton, and nutrients and their temporal and spatial distribution. Insights from this work have the potential to support regional management strategies to assess and improve conditions of water quality endpoints of [DO], chlorophyll *a,* and water clarity to encourage the recovery of seagrass beds.

## 2. Methods

### 2.1. – Site description and monitoring data

**Fig. 1.**
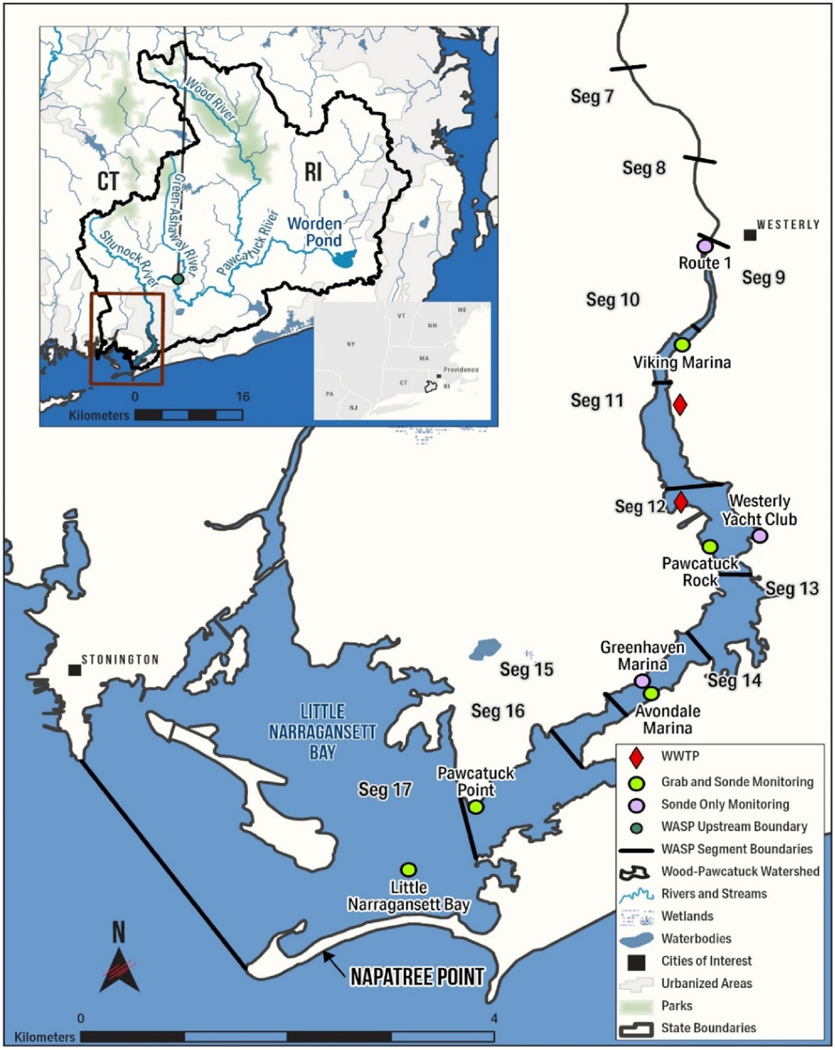
Wood-Pawcatuck Watershed and WASP model domain. (a) Land use information and major tributaries. The Wood-Pawcatuck Watershed boundary forms the HSPF watershed model domain (Lupo et al., 2022). (b) Lower section of the WASP model domain (Segments 7 to 17). Upper left inset map provides the full WASP model domain, the green circle representing the upstream boundary. Black lines designate model segment boundaries, the green dots for monitoring sites with grab and sonde data, the purple dots for sites with only sonde monitoring, and the red diamonds represent the waste-water treatment plants (WWTP).

The PRE is a small, shallow system on the border of Connecticut (CT) and Rhode Island (RI), USA (Fig. 1). Beginning in Worden’s Pond (South Kingstown, RI), the Pawcatuck River runs 56 km to LNB (between Westerly, RI and Stonington, CT). Average depth is 1.5–2.2 m upstream by Viking Marina and increases to 1.9–3.2 m downstream by LNB. The Wood-Pawcatuck Watershed has a drainage area of 283 km^2^ (Connecticut Department of Energy and Environmental Protection, 2020b). Concentrations of particulate organic carbon (POC), dissolved organic carbon (DOC), and total organic carbon (TOC) from the watershed increase with increased river discharge (Fulweiler et al., 2003). Land use consists of forest (58%), wetlands (17.5%), agriculture (8.5%), and urban/suburban communities (12%) (Connecticut Department of Energy and Environmental Protection, 2020b; Heffner, 2007). Most of the population within the watershed resides in rural communities or small towns. Westerly, RI and Stonington, CT are two larger communities surrounding the southern Pawcatuck River. There are two waste-water treatment plants (WWTPs, see Fig. 1), which release into the Pawcatuck River (Connecticut Department of Energy and Environmental Protection, 2020b). WWTP CT0101281 (Stonington, CT) is a secondary treatment facility with denitrification and year round ultraviolet disinfection (Connecticut Department of Energy and Environmental Protection, 2020a). WWTP RI0100064 (Westerly, RI) is a secondary treatment facility with chlorine disinfection (RIDEM, 2021).

Continuous (15-min intervals) sonde data and grab samples were collected across several years with locations designed to capture longitudinal, vertical, and lateral spatial variation within the system (Fig. 1). See the Supplementary Materials (SM), Section 3 for further details. Observed data include: salinity (SAL, ppt), water temperature (WT, °C), depth (DEP, m), dissolved oxygen (DO, mg/L), chlorophyll *a* (chl *a*, μg/L as chl *a*), ammonium (NH_4_, μg/L as N), nitrate (NO_3_, μg/L as N), dissolved inorganic phosphorus (DIP, μg/L as P), total nitrogen (TN, μg/L as N), total phosphorus (TP, μg/L as P), and total suspended solids (TSS, μg/L).

### 2.2. – Model description

The WASP model domain consists of 17 segments representing the last 16 km of the PRE. The results of this study focus on Segments 9–17; the last 10 km where observed data are available (Fig. 1). Downstream sites refer to those in Segments 17–16, mid-stream sites are in Segments 15–12, and upstream sites are in Segments 11–9. Additional details on segment dimensions are provided in the SM (Section 1). The PRE model is constructed using HSPF and WASP, which were selected due to their wide use and applicability for environmental study, management, and TMDL development. HSPF is used to simulate watershed hydrology and loads. This model provides Pawcatuck River discharge, upstream boundary conditions, boundary conditions of the Shunock River (WASP Segment 5), watershed loads into every WASP segment, and WWTP loads. Further details are provided in SM Section 2.2. WASP is a numerical framework for spatial and temporal simulation of water quality. Our model design incorporates 5 modules within WASP: Dynamic Wave hydrodynamics, Water Temperature and Light, Advanced Eutrophication, Macroalgae, and Sediment Diagenesis. Information on each of these modules can be found in SM Sections 2.3 through 2.7. All simulated state variables, abbreviations, units, and initial conditions are listed below in Table 1. For this paper, model outputs focus on salinity (ppt), water temperature (°C), total nitrogen (mg/L as N), total phosphorus (mg/L as P), CBOD (mg/L), phytoplankton (μg/L as chl *a*), DO (mg/L), and macroalgae (gDW/m^2^).

### 2.3. – Model application

The model simulation ran from January 1, 2016, through July 31, 2020, with an average timestep of 0.011 min (0.66 s) and model output every 0.05 days (1.2 h). A variety of model outputs were used to assess water quality endpoints of DO, chlorophyll *a*, water clarity to aid in seagrass restoration. To predict the impact of observed macroalgae (*Cladophora* spp.) on water quality in LNB, and avenues by which macroalgae may limit seagrass growth, a simulation comparison was performed with the absence of macroalgae as a state variable in Segment 17 (LNB). Model outputs, such as WASP’s internally calculated light, nitrogen, and phosphrous phytoplankton growth limitations, were compared with and without macroalgae to assess the simulated impact on other producers. Additional scenarios evaluated how simulated [DO] was affected by the parameterization of phytoplankton growth, CBOD decay, and sediment diagenesis rate constants. Rate constants were divided by ten and multiplied by a factor of ten to provide high and low scenario cases. These scenarios allowed us to explore the relative impact of each DO-affecting process and their temporal and spatial variability. Each parameter was evaluated independently with other parameters held constant at calibrated values. DO loss due to CBOD decay, SOD, and phytoplankton respiration were also calculated to quantify the impact of DO-removing processes throughout the system.

## 3. Results

### 3.1. – Hydrodynamics – salinity and water temperature

**Fig. 2.**
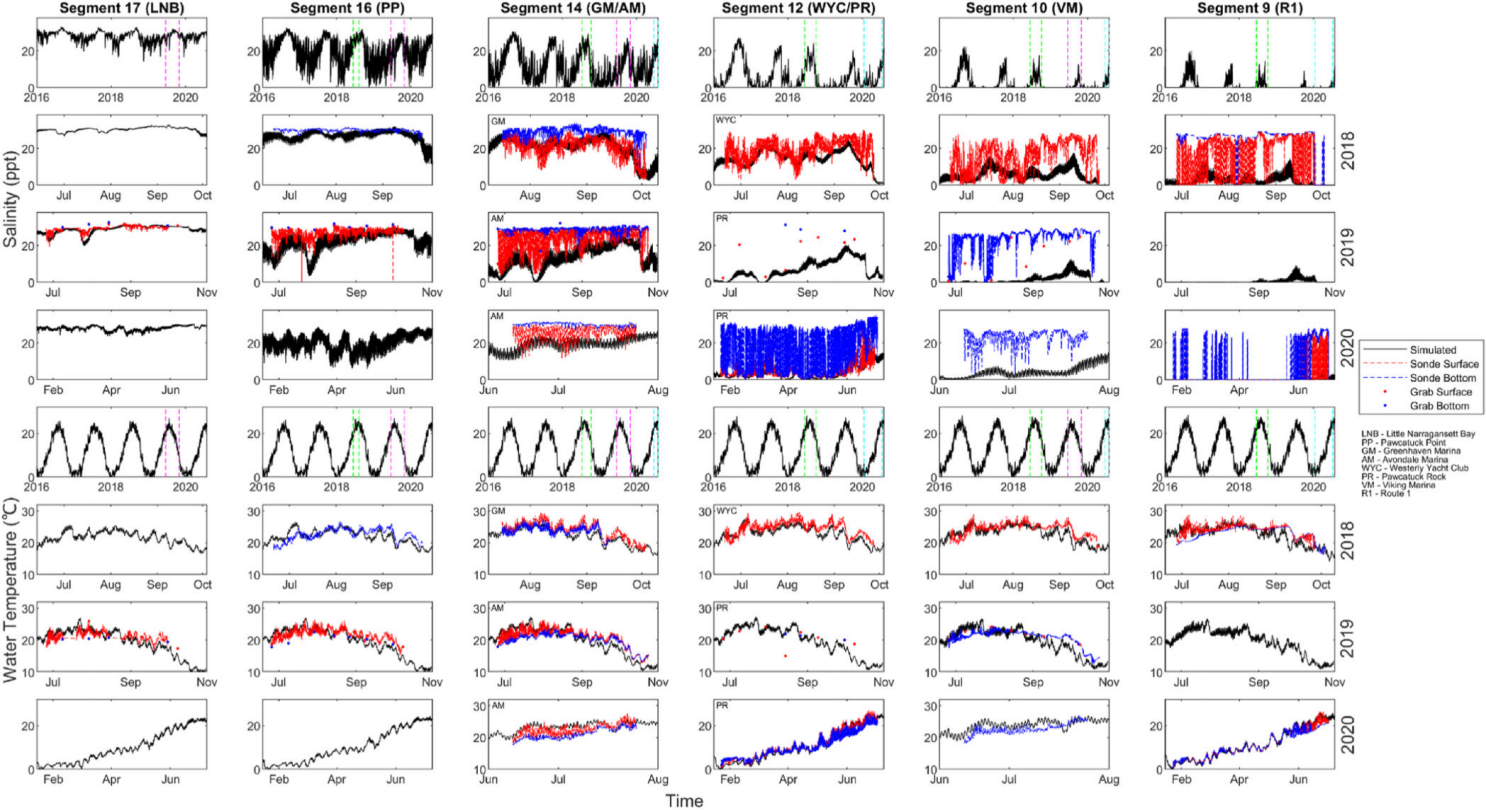
Simulated and observed values of salinity and water temperature. Full simulated results (rows 1, 5) and smaller time periods of observed data (rows 2–4, 6–8). Dashed green lines indicate the zone presented in the second and sixth row (2018), dashed magenta lines for the third and seventh row (2019), and dashed cyan lines for the fourth and eighth row (2020). Site abbreviations are defined below the legend.

Observed salinity concentrations suggested variability in the longitudinal, vertical, and lateral directions (Fig. 2). Near the surface (“surface waters”), there was an increasing gradient moving from upstream to downstream with a mean of 15.7 ppt (Viking Marina, VM) to 29.2 ppt (LNB). The variability and range of salinity decreased moving downstream from 0.2 to 28.5 ppt (VM) to 19.3–32.4 ppt (LNB). For water near the sediments (“bottom waters”), the observed gradient was less severe with means of 23.7 ppt (VM) and 29.9 ppt (Pawcatuck Point, PP). The range of salinity in the bottom waters decreased from 0.06 to 29.7 ppt (VM) to 24.5–31.6 ppt (PP). Sites with both surface and bottom data such as Greenhaven Marina (GM) displayed means of 20.5 ppt and 29.4 ppt respectively. Lower surface means, as well as larger ranges of 2.9–30.0 ppt compared to 9.8–34.0 ppt, demonstrated consistent stratification with depth.

Sites within the same WASP segment suggested lateral variation in the surface waters. Despite the proximity of mid-stream sites, observed data had different means and ranges of salinity concentration. Although not a direct comparison due to different sampling years, mean salinity of 24.8 ppt (Avondale Marina, AM) in 2019 was higher than 20.5 ppt (GM) in 2018. In 2018, mean surface salinity of 19.4 ppt at Westerly Yacht Club (WYC) exceeded that of 10.8 ppt at Pawcatuck Rock (PR). The respective ranges were 2.2–29.9 ppt and 1.8–23. ppt.

The model predicted the decreasing longitudinal salinity gradient, the distance of the salinity front reaching far upstream by Route 1, and the observed seasonal shift with lower predictions in winter months compared to those in summer. The model did not capture the large degree of observed variability in salinity, particularly upstream, with a smaller range of 0.0–17.6 ppt at Segment 10 compared to 0.0–29.7 ppt (VM). The model did not predict large swings in observed data that occur on a smaller temporal scale. Being 1D, the model is not designed to capture vertical or lateral variation. Simulations generally predicted observed conditions better at the surface, demonstrated with a simulated mean of 21.0 ppt (Segment 14) compared to 20.5 ppt at the surface and 29.4 ppt at the bottom (GM). Additionally, the model predicted the trends of observed data more accurately from sites located on the west side of the river compared to the east side. Simulated values predicted the observed range and mean at PR more accurately than those of WYC in 2018. See SM Section 5.1 (Table S6) for additional statistics.

Observed water temperature in Fig. 2 portrayed seasonal variation, reaching lows of 0 °C and highs of 29 °C. Observed temperatures exhibited a narrow range of small-scale temporal variability, with minimal spatial variability, in either the lateral, longitudinal, or vertical direction. Similarity of observed water temperatures throughout the system indicated that they are not impacted by stratification and variable freshwater inflow to the same extent as salinity. The model simulated the observed range and mean of water temperature effectively throughout the model domain.

### 3.2. – macroalgae

**Fig. 3.**
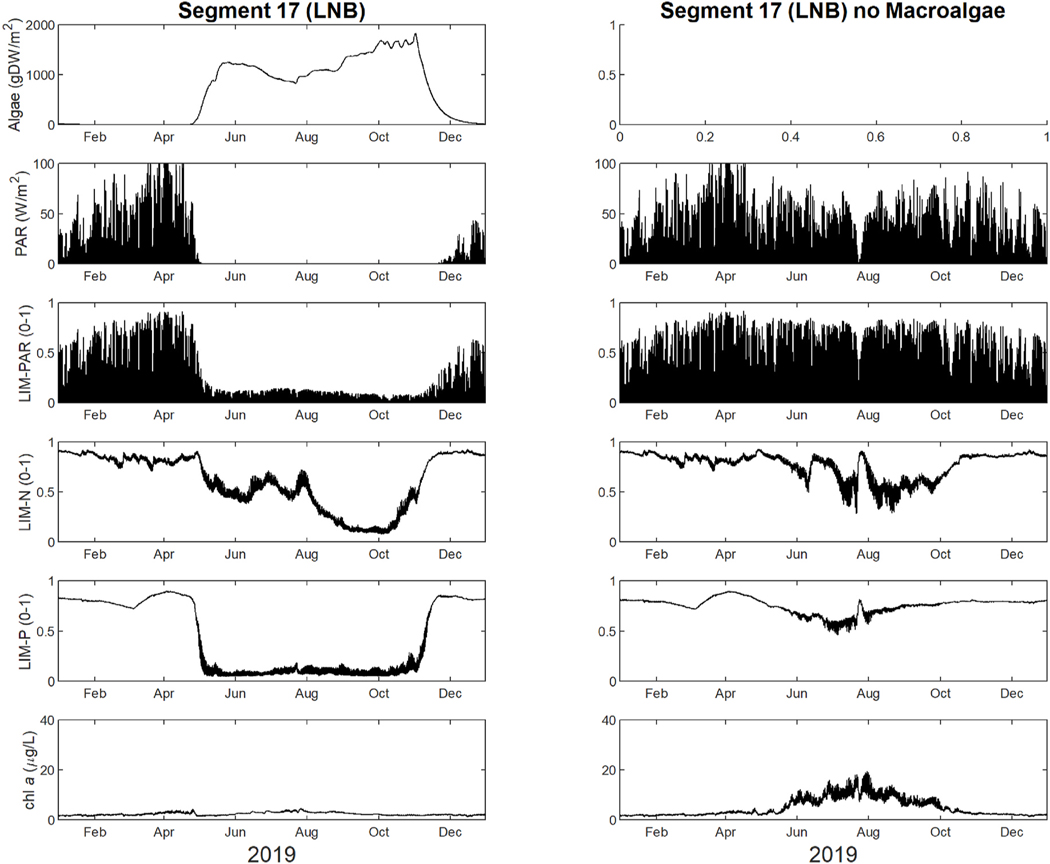
Model simulations with (left) and without (right) macroalgae in Little Narragansett Bay (LNB, Segment 17). From top to bottom, the rows show: macroalgae biomass concentration, light intensity as photosynthetically active radiation (PAR), phytoplankton light growth limitation (LIM-PAR), phytoplankton nitrogen growth limitation (LIM-N), phytoplankton phosphorus growth limitation (LIM-P), and phytoplankton concentration (chl *a*). Simulations present data for the entire year of 2019.

Mechanistic inference of macroalgae simulations suggested that macroalgae blooms began rising in mid-spring (~1100 gDW/m^2^), sagged in the summer (~770 gDW/m^2^), and peaked again in the fall (~1800 gDW/m^2^) before dropping in winter (Fig. 3). During the span of these blooms, the model predicted that no light (photosynthetically active radiation - PAR) reaches the bottom. Conversely, when macroalgae was not simulated, PAR reached the bottom, although it was limited by the presence of phytoplankton. The predicted PAR extinction coefficient ranged from 6 to 17 (1/m) in the presence of macroalgae and 0–1.5 (1/m) without. The WASP model calculates light extinction for every segment at every timestep as a function of TSS, chl a, DOC, and macroalgal shading (See SM Section 2.4). Light availability can also be assessed via WASP’s calculated phytoplankton light limitation factor (LIM-PAR). Phytoplankton limitation factors are calculated internally by WASP and range from a value of 0–1 with 0 being complete limitation and 1 being no limitation. The mean simulated LIM-PAR increased from 0.1 to 0.2 with the removal of macroalgae. Without the presence of macroalgae, PAR in the summer months was highly dynamic. Diel solar radiation alongside larger phytoplankton blooms caused daily and seasonal shifts.

Simulations also illustrated that the nitrogen and phosphorus availability change without macroalgae in the system. The model suggested that the inclusion of macroalgae increased the limitation of phytoplankton growth via nutrient depletion (Fig. 3). The mean phytoplankton nitrogen growth limitation (LIM-N) increased from 0.6 to 0.7 without the presence of macroalgae. Once the macroalgae blooms begin, simulations suggested that the LIM-N had a decreased daily range and variability. The simulated phytoplankton phosphorus growth limitation (LIM-P) showed greater change as the mean value increased from 0.4 to 0.7 without the presence of macroalgae. For phytoplankton, simulations suggested that once macroalgae increased, phytoplankton concentrations decreased and remained low. Simulated mean [chl *a*] in LNB was predicted to be more than doubled (2.4–4.9 μg/L) in the simulation with no macroalgae. The simulated maximum [chl *a*] of 19.4 μg/L is higher without macroalgae compared to 4.5 μg/L.

### 3.3. – dissolved oxygen and phytoplankton

**Fig. 4.**
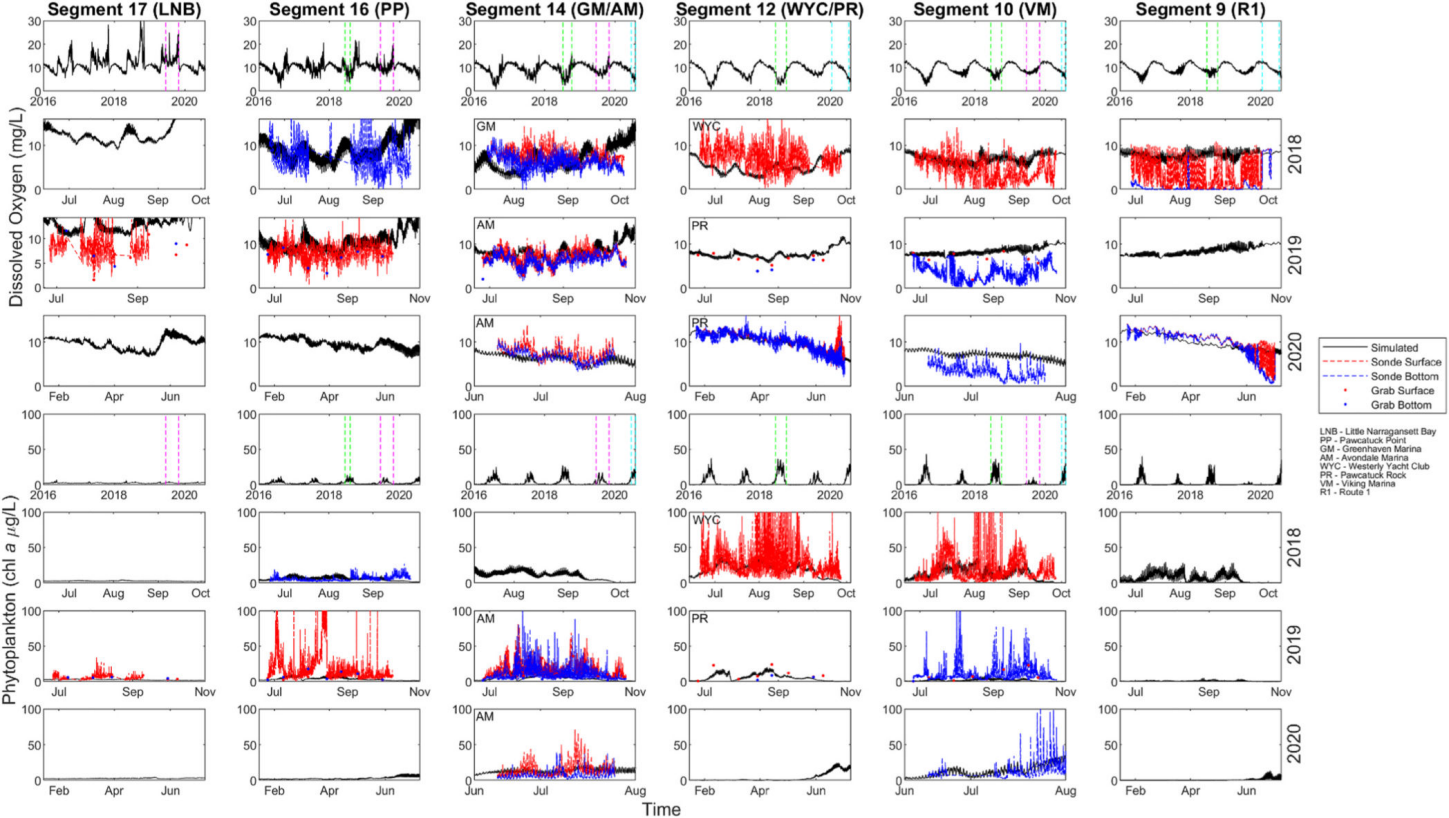
Simulated and observed values of dissolved oxygen and phytoplankton. Full simulated results (rows 1, 5) and smaller time periods of observed data (rows 2–4, 6–8). Dashed green lines indicate the time period presented in the second and sixth row (2018), dashed magenta lines for the third and seventh row (2019), and dashed cyan lines for the fourth and eighth row (2020). Site abbreviations are defined below the legend.

Observed [DO] was highly variable, both spatially and temporally (Fig. 4). The observed data showed there was a longitudinal gradient present. Mean surface [DO] generally decreased moving upstream from 8.3 mg/L (LNB) to 4.7 mg/L (VM). Despite the decreasing mean, the range of [DO] remained large with values of 1.9–17.0 mg/L (LNB) and 0.0–14.1 mg/L (VM). At sites where surface and bottom data are available, observed [DO] also illustrated a vertical gradient. The surface [DO] was consistently higher than bottom [DO], such as 8.0 mg/L compared to 6.4 mg/L (GM). Unlike the salinity of this system, the [DO] did not show any indication of lateral variation. Means of 7.5 mg/L (WYC), 9.9 mg/L (PR, surface), and 6.8 mg/L (PR, bottom) as well as similar observed ranges illustrated the lack of lateral variation. Observed [DO] also showed variability daily and seasonally. Large, rapid swings in [DO] were seen throughout the system and were mostly evident in the surface waters of down-to mid-stream sites. Seasonally, observed [DO] was higher and less variable in the colder months. At Pawcatuck Rock and Route 1 in the year 2020, [DO] in the winter and spring was less variable and showed a higher mean until falling around April–May.

Generally, the simulated mean [DO] decreased moving from downstream (10.3 mg/L, Segment 16) to the mid-stream segments (4.8 mg/L, Segment 12) and then increased moving further upstream (6.3 mg/L, Segment 10). Simulated [DO] also suggested less diel variation and smaller ranges upstream of 4.7–8.1 mg/L (Segment 10) compared to 4.3–20.7 mg/L (Segment 17). Variability of modeled [DO] also shifted with seasonality. Simulated [DO] had a higher mean and smaller range in the winter months.

Observed mean phytoplankton, illustrated by [chl *a*], was highest in the PP and mid-stream areas (Fig. 4). In the summer of 2019, PP had the highest mean [chl *a*] of 17.9 μg/L and a range of 0–212.7 μg/L. Midstream mean [chl *a*] was slightly lower at 14.4 μg/L (AM), decreased further upstream at 9.3 μg/L (VM), and was lowest downstream at 6.0 μg/L (LNB). In 2018, the highest [chl *a*] mean was 30.0 μg/L (WYC) compared to 18.2 μg/L (VM) and 5.6 μg/L (PP). Simulations predicted spatiotemporal variation in the segments of highest mean [chl *a*]. In 2019, the highest mean concentration was 5.8 μg/L (Segment 14) and in 2018 it was 18.2 μg/L (Segment 10). The model properly predicted lower concentrations downstream in 2019 with the observed and simulated mean of 6.0 μg/L and 2.9 μg/L respectively. The continuous data at the surface and bottom of various locations portrayed large, sporadic peaks in [chl *a*] followed by rapid drop offs. Both the simulations and observed grab samples collected during this time did not reflect these large swings. This was represented in simulations by smaller, lower ranges without the daily variability.

Spatiotemporal variation in simulated water quality components (TN, TP, phytoplankton, DO) was also presented in Figs. S8–S12. These figures presented the daily concentrations (12:00) for each year of the model simulation in WASP segments 10–17. These figures emphasize the decreased total nitrogen concentration ([TN]) moving downstream, low total phosphorus concentration ([TP]) throughout the system, the spatiotemporal variation in phytoplankton blooms, and the corresponding zones of hypoxia that developed.

## 4. Discussion

### 4.1. – Hydrodynamics – salinity and water temperature

Observed data suggest heterogeneous salinity gradients in the longitudinal (downstream to upstream), vertical, and lateral directions. Longitudinally, the surface waters downstream have more constant, high mean salinity with small ranges, whereas ranges and variability in salinity increases upstream. Grab samples in LNB also suggest the area is well-mixed. Observed data within mid-stream and upstream sites suggest that the PRE is a vertically complex environment as stratification peaks with increased freshwater inflow in the spring months. This phenomenon has been observed in other complex estuaries such as Gyeongin Port, South Korea, where stratification peaked during the rainy season (Zhu et al., 2020). There, vertical mixing was also impacted by the tidal boundary as the decreased turbulent energy during neap tide increased stratification compared to spring tide (Zhu et al., 2020). What remains unclear in the PRE is if the salinity wedge is spatially constant with freshwater flowing on top of it, or if the wedge moves upstream and downstream with tidal and freshwater interactions. Additionally, lateral variation is suggested by differences in observed ranges and means, as well as higher R^2^ values, for comparisons of simulated data to sampling sites on the west side of the river than on the east. This may be due to the navigational channel that runs through the west side of the river (NOAA Office of Coast Survey, 2014). This may create a preferential flow path through the river, resulting in mixing and advection of salinity. The east side may also lack sufficient flow to create mixing in the water column. These salinity gradients have the potential to create zones of ecological variability in the system. Studies have observed ecological changes along salinity gradients in estuaries such as changes in biological diversity and community structure (Muylaert et al., 2009), benthic metabolism (Hopkinson et al., 1999), and nutrient cycling (Osborne et al., 2015).

Numerical dispersion may explain the under-prediction of mean upstream salinity. Solving the dynamic wave equations requires a small timestep, which can introduce artificial mixing, diminishing the strength of the advective front (Gatto et al., 2021). Additionally, the shallow nature of the system, particularly by the estuary inlet (around Segments 9–10), may make this area more susceptible to changes in salinity and water quality via changes in depth. Our 1D model does not capture the observed vertical and lateral variation. To reproduce this spatial complexity, it would require a three-dimensional (3D) hydrodynamic model. Similar modeling initiatives have taken place in complex systems such as Shenandoah River (Mbuh et al., 2019), the Neuse River Estuary, USA (Wool et al., 2003), Cedar Creek Reservoir, USA (Ernst and Owens, 2009), and Tampa Bay, USA (Wang et al., 1999). Unlike these sites, however, the PRE is small and shallow in comparison, which may limit the applicability of a 3D model. The model simulated water temperature better than salinity. Water temperature may be more vertically homogeneous due to the shallow nature of the system, as well as the similar water temperature profile (R^2^_=_0.93) at each boundary. A benefit of this model design is simulating both salinity and water temperature due to the observed difference in their physical distribution.

### 4.2. – macroalgae

Due to the availability of observed data, only simulations of macroalgae are presented for mechanistic inference. Despite this, qualitative observations of macroalgae within LNB have recorded areas with up to 100% surface coverage of *Cladophora* spp. (Connecticut Department of Energy and Environmental Protection, 2020b). Simulations indicate that macroalgae begin to bloom in early spring, experience a sag in biomass in the summer, and then peak again in the fall before dropping in the winter. This seasonality of *Cladophora* growth dynamics in temperate zones has been observed in other systems such as the Great Lakes (Higgins et al., 2008b). Growth studies and models of macroalgae present several hypotheses explaining the summer sag of macroalgae such as nutrient depletion (Fujita et al., 1989; Mantai, 1974), self-shading (Higgins et al., 2006), or high ambient temperatures (Whitton, 1970). These factors, or a combination of such factors (Canale and Auer, 1982), can lead to metabolic imbalance, cell deterioration, and eventual breakage of macroalgae (Higgins et al., 2008a).

Once the macroalgae bloom, simulations suggest that there is a noticeable impact on various aspects of water quality (Fig. 3). Of particular interest is the limitation of phytoplankton via macroalgae, and how that may relate to eelgrass limitation in LNB. The WASP model internally calculates phytoplankton growth limitation factors displayed in Fig. 3 and S7. WASP limitation factors have been used effectively to assess nutrient concentration and the impact on phytoplankton growth in studies such as the EFDC-WASP modeling initiative in the Nakdong River, South Korea (Seo et al., 2012). Simulations suggest that macroalgae within LNB limits phytoplankton growth via excessive shading and nutrient depletion. Simulated phytoplankton is severely diminished in the presence of macroalgae blooms, whereas the population is more than doubled without macroalgae. After blooms grow in early spring, photosynthetically active radiation (PAR) is heavily reduced, and the phytoplankton light growth limit (LIM-PAR) is also more severe. Light limitation via macroalgae was also observed in Waquoit Bay, USA, where the eelgrass *Zostera marina* was severely limited by the lack of light (Hauxwell et al., 2003). Further, our simulations suggest that large blooms of macroalgae outcompete phytoplankton via depletion of nitrogen and phosphorus. Observations from mesocosm experiments suggest that macroalgae blooms can limit the growth of phytoplankton via competition for nutrients in enriched environments (Fong et al., 1993). Additional studies show that light limitation, species interactions such as competition with other primary producers, and alteration of biochemical cycles via eutrophication inhibit eelgrass and phytoplankton growth (Short et al., 1995; Dodds and Gudder, 1992)).

Our simulation of macroalgae, as well as in the development of a macroalgae model for Greenwich Bay, USA (Brush and Nixon, 2010), emphasize the importance of incorporating macroalgae in future water quality studies. Various macroalgae species play important roles in regulating the water quality and ecosystem processes of a system (Brush and Nixon, 2010). Additionally, the complexity of these modules highlights the need for further studies on the biology of these organisms to constrain and improve model formulations.

### 4.3. – dissolved oxygen and phytoplankton

Simulated and observed data suggest that the spatial and temporal complexity of the hydrodynamics and ecological processes have a large impact on [DO]. Spatially, observed and simulated mean [DO] decrease moving upstream, however simulated [DO] increase upstream of Segment 11, while observed [DO] remains low. The processes causing the continuation of the observed, decreasing gradient of [DO] upstream by Viking Marina is not clear and merits further investigation. Simulations suggest that hypoxia begins around June, peaks in intensity during the mid-summer, and weakens in October to November (Figs. S10–S14). Mean [DO] decrease from downstream into the mid-stream may be due to nutrient input and phytoplankton concentrations. Larger phytoplankton concentrations, as well as WWTP inputs (Segments 11 and 12), could cause a large range and lower mean of [DO] due to settling and decomposition of POM. This, along with the temporal variations from seasonality and tidal action, correspond with predicted zones of hypoxia. CBOD inputs from WWTPs may also result in a higher proportion of DO being removed from the water column (Fig. S6). In additional model scenarios (Fig. S15), the parameterization of rate constants for phytoplankton growth, CBOD decay, and sediment diagenesis of organic carbon were increased and decreased by a factor of ten to assess their relative impact on [DO] in the PRE. These simulations suggest that CBOD could be the largest driver of hypoxia throughout the PRE. The importance of CBOD has been observed in additional numerical modeling studies such as that in the Rappahannock River, USA. CBOD was the most important DO-consuming process in the water column and water column oxygen demand was equally as important as SOD (Park et al., 1996). The processes causing the continuation of the observed, decreasing gradient of [DO] upstream by Viking Marina is not clear and merits further investigation.

Differences between observed surface and bottom [DO] emphasized the importance of SOD as a primary driver of hypoxia in the bottom waters. Observed [DO] in the bottom waters are consistently lower than those of the surface, and hydrodynamics (Fig. 2) emphasize the presence of stratification. These suggest that SOD is a localized process where the salinity wedge may act as a boundary layer, creating a decrease in [DO] of the bottom waters. Studies of SOD in other stratified environments such as the Changjiang Estuary (East China Sea) have identified this phenomenon as a major sink of DO in waters below the pycnocline (Zhang et al., 2017). The additional model scenarios investigating parameterization (Fig. S6 and Fig. S15) did not suggest a large effect of SOD decay, which may be driven by the 1D design. Ecosystems with historical pollution via excessive nutrient input have also identified SOD as an important factor because of the abundance of organic material in the sediments (MacPherson et al., 2007). This vertical complexity, as well as horizontal variation in the impact of DO processes, indicate that future development of a 3D model may provide more insight towards these processes and the scale of their spatial variation.

Assessment of DO in this system was limited by available observed data and unknown parameterization values. Sediment Diagenesis and Advanced Eutrophication are complex ecological modules that are non-linear, require a large amount of data to parameterize, and consist of many governing processes. Available information regarding observed SOD and diagenesis rates, phytoplankton and macroalgae growth and respiration, and CBOD concentration and decay rates limits our ability to calibrate the model with proper constraint. Increased information on these processes would improve model development and system understanding to aid reductions of hypoxia throughout the PRE and generally improve our understanding of processes in similar small, shallow estuaries.

Observed phytoplankton concentration ([chl *a*]), show high peaks followed by rapid decreases. Spatially, the mean [chl *a*] was highest from Pawcatuck Point to the mid-stream sites, slightly lower upstream, and lowest downstream. This spatial trend also aligns well with the impact of the WWTP’s. The nitrogen and phosphorus load from these systems may enable the highest phytoplankton growth in the mid-stream area. Simulations predict that TN concentrations decrease moving downstream, and phytoplankton blooms peak in the mid- and upstream area (Figs. S10–S14). The peaks in nutrient loads from WWTP’s relate to phytoplankton growth patterns and the corresponding hypoxia. The model predicted the spatial variation and mean [chl *a*] but not the dynamic peaks. Studies have identified several mechanisms which may cause large spatial and temporal variation of phytoplankton. Study of San Francisco’s South Bay suggested mechanisms of temporal variability such as the short-term variability of tides, light availability, residence time, zooplankton grazing, horizontal dispersion, and sediment to water column exchanges of microalgae (Cloern et al., 1985). Assessment of the Neuse River Estuary indicated that nutrient (specifically nitrogen) availability as well as nutrient type may influence phytoplankton biomass and community structure (Cira et al., 2016). While simulations suggested macroalgae limit phytoplankton downstream in LNB, a potential explanation of lower concentrations upstream may be the distribution of dissolved organic carbon in the PRE. Sampling studies of the PRE have found that variability in the light extinction was largely explained by DOC concentration, which may limit the accumulation of phytoplankton biomass (Doering et al., 1994).

### 4.4. – Environmental management implications

Insights from this study have environmental implications which have the potential to aid in the management and restoration of similar small, shallow coastal embayments. The size and hydrodynamics of the PRE create features which resemble both small, freshwater systems as well as larger, coastal bays. Typically found in freshwater rivers, the PRE displays a [DO] sag due to CBOD inputs which resembles that of the classic Streeter-Phelps equation. CBOD inputs by the WWTP’s cause a sag in the observed [DO] by Avondale Marina which recovers further downstream and into the Bay. This phenomenon has been observed in many other freshwater streams such as the Harsit Stream which flows into the Black Sea (Nas and Nas, 2009). The PRE also exhibits fluctuations in DO caused by phytoplankton growth and respiration and SOD which resembles that of larger systems. Study of shallow estuaries like the PRE have indicated that benthic metabolism from submerged aquatic vegetation as well as subtidal sediments can dominate DO cycling (Ganju et al., 2020; Testa et al., 2022). Further, unlike deeper systems where oxygen depletion is largely seasonal, shallow ecosystems can experience local diel cycling because of increased benthic metabolism or water-column respiration due to phytoplankton biomass (Testa et al., 2022; Tyler et al., 2009). Evaluation of Narragansett Bay also found that daily fluctuations in DO were driven by phytoplankton activity whereas the general trends of DO were more likely driven by sediment influence (Knightes, 2023). These active processes in the PRE suggest that there are multiple avenues that should be considered when developing management strategies to restore this ecosystem.

The complexity demonstrated in the PRE also warrants the need for increased observation of similar ecosystems. The acquisition of observed data, with spatial resolution in three directions, enabled the understanding of the various processes affecting DO and water quality. Further, the acquisition of continuous sonde data alongside discrete data emphasized the diurnal variation and large range of water quality parameters which would have been previously missed. In Narragansett Bay, the collection of sonde data also suggested that the current WASP framework may not be structured to simulate the hourly variability that was observed (Knightes, 2023).

## 5. Conclusion

In this study, we combined an analysis of observed data with a 1D, numerical, contaminant fate and transport model to improve our understanding of the temporal and spatial variations in water quality throughout the PRE. This work is relevant to environmental managers by highlighting multiple pathways by which eutrophication can cause hypoxia and seagrass loss. Our model suggests the importance of addressing a variety of processes (phytoplankton growth, SOD, CBOD decay, shading, etc.) when creating management strategies for the PRE and similar, eutrophic ecosystems.

This environment has a history of excessive nutrient loading and eutrophication, resulting in zones of hypoxia, algal growth, and decreased water clarity. The hydrodynamics of the system was reflected in the spatial and temporal variability in salinity concentrations. The observed variability in salinity patterns also had implications for water quality throughout the system. The Dynamic Wave module was effective at simulating the down- and midstream hydrodynamics, but undersimulated surface and bottom salinity upstream. For management strategies focused on the downstream and midstream zones, the use of Dynamic Wave hydrodynamics may be sufficient. Macroalgae had a large impact on water quality downstream via shading and nutrient depletion which limited phytoplankton growth, supporting the importance of including macroalgae as a simulated variable. Improving simulation accuracy and parameterization of macroalgae will allow for investigating management strategies to minimize growth and improve the understanding of macroalgae on similar systems. The introduction of loads from headwaters and WWTPs result in [TN] decreasing moving downstream, low [TP] throughout the system, and the rapid growth of large phytoplankton blooms. Model simulations suggested that the phytoplankton is mostly limited by phosphorus throughout most of PRE and is only similarly nitrogen limited in LNB. [DO] was also dynamic likely because of phytoplankton blooms and CBOD loads from WWTPs. CBOD decay may be the primary driver of low [DO] in the water column, and SOD may be the primary driver of hypoxia in the stratified, benthic waters. The DO loss due to phytoplankton respiration was not large but increased moving upstream.

Alongside supporting regional management and restoration, future work will continue in the PRE to improve our knowledge and governing processes of this and other similar ecosystems. While hydrodynamic model simulations performed well from LNB to the mid-stream area, the spatial variability that is present may merit the use of a 3D hydrodynamic model. This would provide greater spatial resolution to feasibly improve simulating the impact of stratification and SOD. However, these models also increase complexity and uncertainty as well as require more resources to construct, parameterize, and calibrate. The benefits and drawbacks of applying a 1D versus 3D model design to simulate this ecosystem is of particular interest moving forward. This work also suggests the need for additional research of producer productivity and behavior in simulating phytoplankton, macroalgae, and SOD to properly parameterize and constrain these complex modules. Advancements in the understanding of this ecosystem and the corresponding modeling work may be a valuable tool in helping protect and restore similar small, coastal embayments.

## Supplementary Material

Supplement1

## Figures and Tables

**Table 1:** A summary table of each state variable in PRE model containing the state variable name, abbreviations, units, and corresponding initial conditions.

WASP State Variable	Abbreviations	Units	Initial Conditions

Salinity	SAL	ppt	0–15
Water Temperature	WT	°C	5
Silt	TSS-S	mg/L	0
Clay	TSS-C	mg/L	0
Dissolved Oxygen	DO	mg/L	12
Amonium	NH_4_	mg/L as N	0.1
Nitrate + Nitrite	NO_3_	mg/L as N	0.1
Particulate Organic Nitrogen	PON	mg/L as N	0.1
Dissolved Organic Nitrogen	DON	mg/L as N	0.1
Dissolved Inorganic Phosphorus	DIP	mg/L as P	0.03
Dissolved Organic Phosphorus	DOP	mg/L as P	0.1
Particulate Organic Phosphorus	POP	mg/L as P	0.1
Carbonaceous Biological Oxygen Demand	CBOD	mg/L	1.5
Particulate Organic Carbon	POC	mg/L	0.1
Phytoplankton	chl *a*	ug/L as chl *a*	0
Macroalgae	MACRO	gDW/m^2^	0–0.5
Macroalgae Internal N	MACRO-N	mg/L	0–0.5
Macroalgae Internal P	MACRO-P	mg/L	0–0.5
Particulate Organic Matter	TOTDE	mg/L	0.5

## Data Availability

Data is shared in Supplementary Material as well as at Mendeley Data, V2, doi: 10.17632/vywyyks4r6.2 Using Monitoring and Mechanistic Modeling to Improve Understanding of Eutrophication in a Shallow New England Estuary (Original data) (Mendeley Data)
