# Supplementary material for: Using monitoring and mechanistic modeling to improve understanding of eutrophication in a shallow New England estuary
Source: J Environ Manage. Author manuscript; Available in PMC 2025 Mar 2. (PMC11091823; doi:10.1016/j.jenvman.2024.120478)
Supplement: Supplement1 [file NIHMS1985679-supplement-Supplement1.docx]

**Using Monitoring and Mechanistic Modeling to Improve Understanding of Eutrophication in a Shallow New England Estuary**

Finnian S. Cashel^a^, Christopher D. Knightes^b^*, Chris Lupo^c^, Traci Iott^d^, Kelly Streich^e^, Corey J. Conville^f^, Timothy W. Bridges^g^, Ian Dombroski^h^

^a^ Oak Ridge Institute for Science and Education Fellow, United States Environmental Protection Agency, Office of Research and Development, Center for Environmental Measurement & Modeling, Atlantic Coastal Environmental Sciences Division, 27 Tarzwell Drive, Narragansett, RI 02882, USA. Email: Cashel.Finnian@epa.gov

^b^ United States Environmental Protection Agency, Office of Research and Development, Center for Environmental Measurement & Modeling, Atlantic Coastal Environmental Sciences Division, 27 Tarzwell Drive, Narragansett, RI 02882, USA. Email: Knightes.Chris@epa.gov

^c^ RESPEC, 3825 Jet Dr, Rapid City, SD 57703, USA. Email: Chris.Lupo@respec.com

^d^ Connecticut Department of Energy and Environmental Protection, Bureau of Water Protection and Land Reuse, 79 Elm Street, Hartford, CT 06106, USA. Email: Traci.Iott@ct.gov

^e^ Connecticut Department of Energy and Environmental Protection, Bureau of Water Protection and Land Reuse, 79 Elm Street, Hartford, CT 06106, USA. Email: Kelly.Streich@ct.gov

^f^ United States Environmental Protection Agency, Region 1, Laboratory Sciences and Applied Science Divisions, 11 Technology Drive, North Chelmsford, MA 01863, USA. Email: Conville.Corey@epa.gov

^g^ United States Environmental Protection Agency, Region 1, Laboratory Sciences and Applied Science Divisions, 11 Technology Drive, North Chelmsford, MA 01863, USA. Email: Bridges.Tim@epa.gov

^h^ United States Environmental Protection Agency, Region 1, Water Division, 5 Post Office Square, Suite 100, Boston, MA 02109, USA. Email: Dombroski.Ian@epa.gov

* Corresponding author, knightes.chris@epa.gov

Table of Contents

[1. Model Domain 1](#_Toc149054042)

[2. Model Description 2](#_Toc149054043)

[*2.1 Hydrological Simulation Program – FORTRAN (HSPF)* 2](#_Toc149054044)

[*2.2 WASP Dynamic Wave* 2](#_Toc149054045)

[*2.3 WASP Water Temperature and Light* 3](#_Toc149054046)

[*2.4 WASP Advanced Eutrophication Module* 3](#_Toc149054047)

[*2.5 WASP Macroalgae* 5](#_Toc149054048)

[*2.6 Sediment Diagenesis Module* 5](#_Toc149054049)

[3. Observed and Input Data 7](#_Toc149054050)

[*3.1 Observed Data* 7](#_Toc149054051)

[*3.2 Input Data* 8](#_Toc149054052)

[4. Parameterization 9](#_Toc149054053)

[5. Calibration and Model Evaluation 17](#_Toc149054054)

[*5.1 Hydrodynamics* 17](#_Toc149054055)

[*5.2 Dissolved Oxygen and Phytoplankton* 22](#_Toc149054056)

[*5.3 Nutrients* 30](#_Toc149054057)

[*5.4 Spatial Variation – Heat Maps* 32](#_Toc149054058)

[*5.5 Parameter Evaluation Effect on Dissolved Oxygen* 37](#_Toc149054059)

[6. Associated Input and Observed Data 41](#_Toc149054060)

[7. References 43](#_Toc149054061)

# **Model Domain**

Table S1 provides the segmentation information from each WASP segment. WASP enables the user to create a customized model domain which reflects the dimensions of the desired observed environment. These dimensions impact the hydrology and corresponding transport of materials within the model. Dimensions of the WASP segments were delineated using [Google Earth's](https://earth.google.com/web) measuring tool. The WASP model domain consists of 17 segments representing the last 16 km of the PRE. Segments 1 to 16 represent the confluence of the Green-Ashaway River and the Pawcatuck River (Segment 1) to Pawcatuck Point (Segment 16). Segment 17 extends to the end of Napatree Point in Little Narragansett Bay (See Figure 1). Segmentation was designed to have similar lengths using morphological features in the river system to assign division points. Bottom elevation was determined using NOAA Nautical Chart 13214 found in Section 6 (NOAA Office of Coast Survey, 2014).

Table S1. Table containing segmentation information from each WASP Segment. Abbreviations of data columns from left to right read: volume (V), length (L), average width (AW), bottom elevation (BE), minimum depth (MD), roughness (R), average depth (AD), initial surface elevation (ISE), depth multiplier (DM), depth exponent (DE), velocity multiplier (VM), and velocity exponent (VE).

| **Segment** | **V (m3)** | **L (m)** | **AW (m)** | **BE (m)** | **MD (m)** | **R** | **AD (m)** | **ISE (m)** | **DM** | **DE** | **VM** | **VE** |
| --- | --- | --- | --- | --- | --- | --- | --- | --- | --- | --- | --- | --- |
| 1 | 7380 | 1230 | 20 | 2 | 0.1 | 0.03 | 0.5 | 2.5 | 0.083 | 0.45 | 2.93 | 0.3 |
| 2 | 5820 | 970 | 20 | 2 | 0.1 | 0.03 | 0.5 | 2.5 | 0.083 | 0.45 | 2.93 | 0.3 |
| 3 | 6360 | 1060 | 20 | 2 | 0.1 | 0.03 | 0.5 | 2.5 | 0.083 | 0.45 | 2.93 | 0.3 |
| 4 | 8062.5 | 1075 | 25 | 2 | 0.1 | 0.03 | 0.5 | 2.5 | 0.083 | 0.45 | 2.35 | 0.3 |
| 5 | 12750 | 850 | 25 | 1.9 | 0.1 | 0.03 | 0.6 | 2.5 | 0.165 | 0.45 | 1.17 | 0.3 |
| 6 | 21315 | 1015 | 35 | 1.9 | 0.1 | 0.03 | 0.6 | 2.5 | 0.165 | 0.45 | 0.84 | 0.3 |
| 7 | 26250 | 1250 | 35 | 1.9 | 0.1 | 0.03 | 0.6 | 2.5 | 0.165 | 0.45 | 0.84 | 0.3 |
| 8 | 21600 | 900 | 40 | 1.9 | 0.1 | 0.03 | 0.6 | 2.5 | 0.165 | 0.45 | 0.73 | 0.3 |
| 9 | 60480 | 1120 | 60 | 1.6 | 0.1 | 0.03 | 0.9 | 2.5 | 0.248 | 0.45 | 0.33 | 0.3 |
| 10 | 142992 | 900 | 160 | 1.507 | 0.1 | 0.02 | 0.993 | 2.5 | 0.273 | 0.45 | 0.11 | 0.3 |
| 11 | 487760 | 1040 | 350 | 1.16 | 0.1 | 0.02 | 1.34 | 2.5 | 0.369 | 0.45 | 0.04 | 0.3 |
| 12 | 723576 | 1180 | 420 | 1.04 | 0.1 | 0.02 | 1.46 | 2.5 | 0.402 | 0.45 | 0.03 | 0.3 |
| 13 | 643968 | 860 | 520 | 1.06 | 0.1 | 0.02 | 1.44 | 2.5 | 0.396 | 0.45 | 0.02 | 0.3 |
| 14 | 636900 | 1100 | 300 | 0.57 | 0.1 | 0.02 | 1.93 | 2.5 | 0.531 | 0.45 | 0.03 | 0.3 |
| 15 | 680000 | 800 | 340 | 0 | 0.1 | 0.02 | 2.5 | 2.5 | 0.688 | 0.45 | 0.02 | 0.3 |
| 16 | 1206072 | 1050 | 480 | 0.107 | 0.1 | 0.02 | 2.393 | 2.5 | 0.658 | 0.45 | 0.02 | 0.3 |
| 17 | 10469550 | 2275 | 2950 | 0.94 | 0.1 | 0.02 | 1.56 | 2.5 | 0.429 | 0.45 | 0 | 0.3 |

# **Model Description**

This section contains information on various modules used to simulate the hydrodynamics and water quality within the PRE model. Additional resources on each module are also provided.

## *2.1 Hydrological Simulation Program – FORTRAN (HSPF)*

The Hydrological Simulation Program – FORTRAN (HSPF) is a comprehensive modeling framework designed to simulate watershed hydrology. HSPF incorporates the simulation of erosion, runoff, contaminant fate and transport from the watershed into receiving waters, and the associated in-stream hydraulic and sediment processes (EPA, 2021). The HSPF model for the Wood-Pawcatuck watershed was developed and implemented by RESPEC (2022). This model simulates the entire watershed and provides total Pawcatuck flow, upstream boundary conditions, subwatershed loads into each associated WASP segment, and the wastewater treatment plant releases for Segments 11 (RI) and 12 (CT). USGS 01118500 (Westerly, RI) was the main gage of HSPF calibration, but additional gages were used and are detailed in Table 2-1 and Figure 2-1 of the Pawcatuck River Watershed Final HSPF Modeling Report (Lupo et al., 2022). The HSPF module requires precipitation, potential evapotranspiration and meteorological data, stream flow, water quality (nutrients, solids, DO), point sources, atmospheric deposition, and other data (i.e., diversions, irrigation, withdrawals) for hydrology and water quality simulations. The input data for the HSPF model, atmospheric forcing functions, as well as additional information are detailed in the Pawcatuck River Watershed Final HSPF Modeling Report (Lupo et al., 2022) which is provided in Section 6.

## *2.2 WASP Dynamic Wave*

Hydrodynamics are simulated using the Dynamic Wave module within the WASP8 framework. Based on the original DYNHYD5 model (Ambrose et al, 1993), the Dynamic Wave flow option has been fully incorporated into WASP8. The Dynamic Wave uses 1D hydrodynamic equations conserving both momentum and volume (Ambrose and Wool, 2017). These equations solve for the propagation of a long wave through a shallow system, using surface elevation and surface slope to calculate flow (Ambrose and Wool, 2017). Dynamic Wave differs from other WASP options in that these equations incorporate variable upstream inflows and downstream tidal heads. This allows for the simulation of flow in both directions and calculates velocity, surface elevation, depth, and volume within a 1D model framework (Ambrose and Wool, 2017). Input data for this module included freshwater inflow and tidal height. This input data can be found in Section 6. Additional information on Dynamic Wave processes and equations can be found at the [WASP8 Stream Transport – Model Theory and User’s Guide](https://www.epa.gov/sites/default/files/2018-05/documents/stream-transport-user-guide.pdf) on the EPA’s WASP Model Documentation site.

## *2.3 WASP Water Temperature and Light*

Water temperature is simulated using heat transfer equations based on the previous WASP HEAT module. Governing equations are based on the conservation of mass and energy (Wool et al, 2020). Variables simulated within this module are temperature, surface heat exchange, sediment heat exchange, and density. Major sources and sinks for full-balance heat exchange are loadings from external sources and the surface heat exchange across the air-water interface (Wool et al, 2020). Inputs for this module are solar radiation (W/m^2^), air temperature (℃), wind speed (m/sec), cloud cover (unitless), and dew point (℃), as well as boundary water temperature profiles (℃). These data can be found in Section 6.

Light intensity is simulated using the Light module in WASP8. Light intensity is simulated in every segment at every time step. WASP breaks total solar radiation into infrared, visible, and ultraviolet (UV) light. Visible and UV light are further divided into 10 wavebands. HSPF calculates light intensity at the ground and water surface by attenuating light through the atmosphere. WASP attenuates light through the water column with calculations based on solid concentration (TSS), chlorophyll *a*, and macroalgae, if present, for each band of light wavelengths (Knightes et al., 2019). Light is used to simulate water temperatures and the growth of phytoplankton and macroalgae.

Additional information on water temperature, light processes, and corresponding equations can be found at the [WASP8 Temperature Model Theory and User's Guide](https://www.epa.gov/sites/default/files/2018-05/documents/heat-model.pdf) and the [WASP8 Light Module and User's Guide](https://www.epa.gov/sites/default/files/2018-05/documents/light-module.pdf) respectively on the EPA’s WASP Model Documentation site.

## *2.4 WASP Advanced Eutrophication Module*

The Advanced Eutrophication module was used to simulate phytoplankton, nitrogen (N), phosphorus (P), carbon (C, CBOD), and dissolved oxygen (DO). Phytoplankton activity and the corresponding environmental impact is determined by specific calibration of phytoplankton parameters. Figure S1 presents the governing processes simulated using the Advanced Eutrophication module. Phytoplankton grow via photosynthesis, which uptakes dissolved organic nitrogen and phosphorus, ammonium, nitrate, phosphate, and releases DO. Phytoplankton respiration releases dissolved organic matter and inorganic nutrients and uptakes DO. These processes occur based on a specific stoichiometric ratio. Parameterization of ratios and rate constants was based on nearby modeling and monitoring work in Narragansett Bay (Dettman, 2020), Niantic River Estuary (Vaudrey et al, 2020), and WASP default values. Phytoplankton death results in the release of particulate organic matter (POM: C, N, and P), which can either settle to the sediments or undergo dissolution to become dissolved organic matter (DOM: N, P, CBOD). Nitrogen and phosphorus-based forms of DOM may mineralize and return to their inorganic forms. Carbon based forms of DOM (CBOD) can oxidize and remove DO from the water column in this process. Inorganic forms of nutrients undergo settling with total suspended solids (TSS), nitrification, and denitrification. Upon settling, POM is processed by the Sediment Diagenesis module (SI Section 2.7), resulting in sediment oxygen demand (SOD), which removes DO from the water column, and benthic flux from the sediments to the water column (P and N). Loadings and boundary concentrations of DO, chlorophyll *a*, and nutrients and organic matter can be found in Section 6. Additional information on the Advanced Eutrophication processes and equations can be found at the [WASP8 Multiple Algae Model Theory and User's Guide](https://www.epa.gov/sites/default/files/2018-05/documents/mpm-user-guide.pdf) on the EPA’s WASP Model Documentation site.


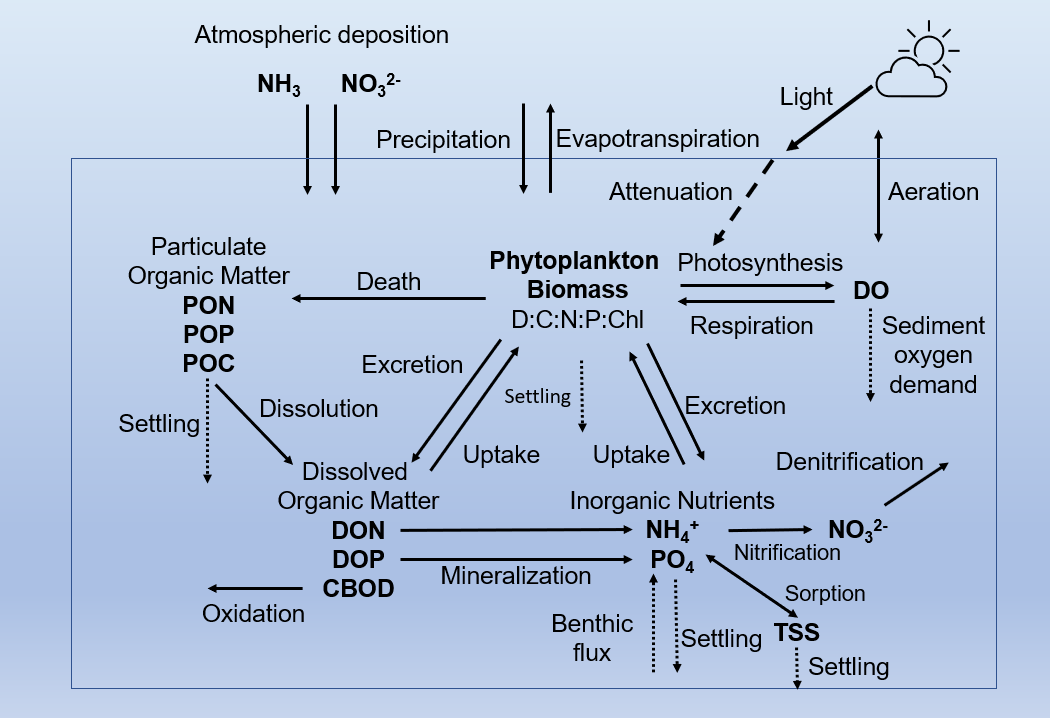


Figure S1. Flow chart illustrating the various reactions taking place in a WASP water quality segment within the Advanced Eutrophication module.

## *2.5 WASP Macroalgae*

The Macroalgae module simulates the processes governing macroalgae. This module was designed to extend upon the previously developed benthic algal model (Martin et al, 2006) to add the ability to simulate different types of macroalgae (Martin et al, 2018). The governing processes of macroalgae are similar to phytoplankton, with photosynthesis, respiration, nutrient uptake and excretion, and death (Figure S1). The PRE model is structured to simulate *Cladophora spp.*, the dominant type of macroalgae found in Little Narragansett Bay (Rollinson et al, 2021). This species is simulated as a floating form that is restricted to Segment 17 (representative of Little Narragansett Bay). Macroalgae is limited to this segment by minimizing (set to zero) or not activating the Macroalgae Drag Transport Function which determines the drag of advective transport. The module is designed to simulate macroalgae that have optimal growth at certain water quality conditions (salinity, water temperature, light, etc.), are capable of luxury uptake of nutrients, and self-shade, which impacts the growth of macroalgae and phytoplankton in the system (Martin et al, 2018). Additional information on the Macroalgae Module processes and equations can be found at the [WASP8 Macroalgae Model Theory and User's Guide](https://www.epa.gov/sites/default/files/2018-05/documents/wasp-macroalgae_manual-v3.pdf) on the EPA’s WASP Model Documentation site.

## *2.6 Sediment Diagenesis Module*

The last module included in the PRE model is Sediment Diagenesis. This module, as well as the inclusion of macroalgae, was first added to the WASP framework in WASP7 in 2011 (Wool et al., 2020). The Sediment Diagenesis module simulates the settling to and decay of organic matter in the sediment layers and calculates sediment oxygen demand (SOD) and benthic nutrient fluxes. This module has two sediment layers per designated overlying WASP surface water segment. The sediments are divided into two layers: a thin, aerobic layer above a thicker, anaerobic layer (Martin and Wool, 2017). Both layers are assumed to be well-mixed. WASP simulates the deposition of particulate organic matter (POM) to the sediments, mineralization of POM in the sediment, the corresponding fluxes between sediment layers and surface water, and the reactions that take place with mineralization products (Martin and Wool, 2017). The collective term of POM is composed of particulate organic carbon (POC), nitrogen (PON), and phosphorus (POP). Upon entering the sediments, POM goes directly into the second layer due to the assumed negligible thickness of the top layer. In the active layer, POM is partitioned into two classes to account for the initial, rapid mineralization of POM and then the following sag in reaction (Martin and Wool, 2017). The first class (POM1) represents a highly reactive class, with a faster decay rate, and the second (POM2) is more stable, with a slower decay rate. Mineralization reactions return organic matter into their inorganic form in an oxygen-demanding process that results in SOD. The products of these reactions (NH_4_^+^, NO_3_^2-^, and PO_4_^3-^) diffuse into the water column as benthic flux. Initial conditions of organic content in the sediment are determined by running a warm-up period simulation to establish quasi-steady state conditions in the sediment, which are then used as initial conditions in the model simulation (Martin and Wool, 2017). Additional information on the Sediment Diagenesis Module processes and equations can be found at the [WASP Sediment Diagenesis Routines Model Theory and User's Guide](https://www.epa.gov/sites/default/files/2018-05/documents/wasp8_sod_module_v1.pdf) on the EPA’s WASP Model Documentation site.


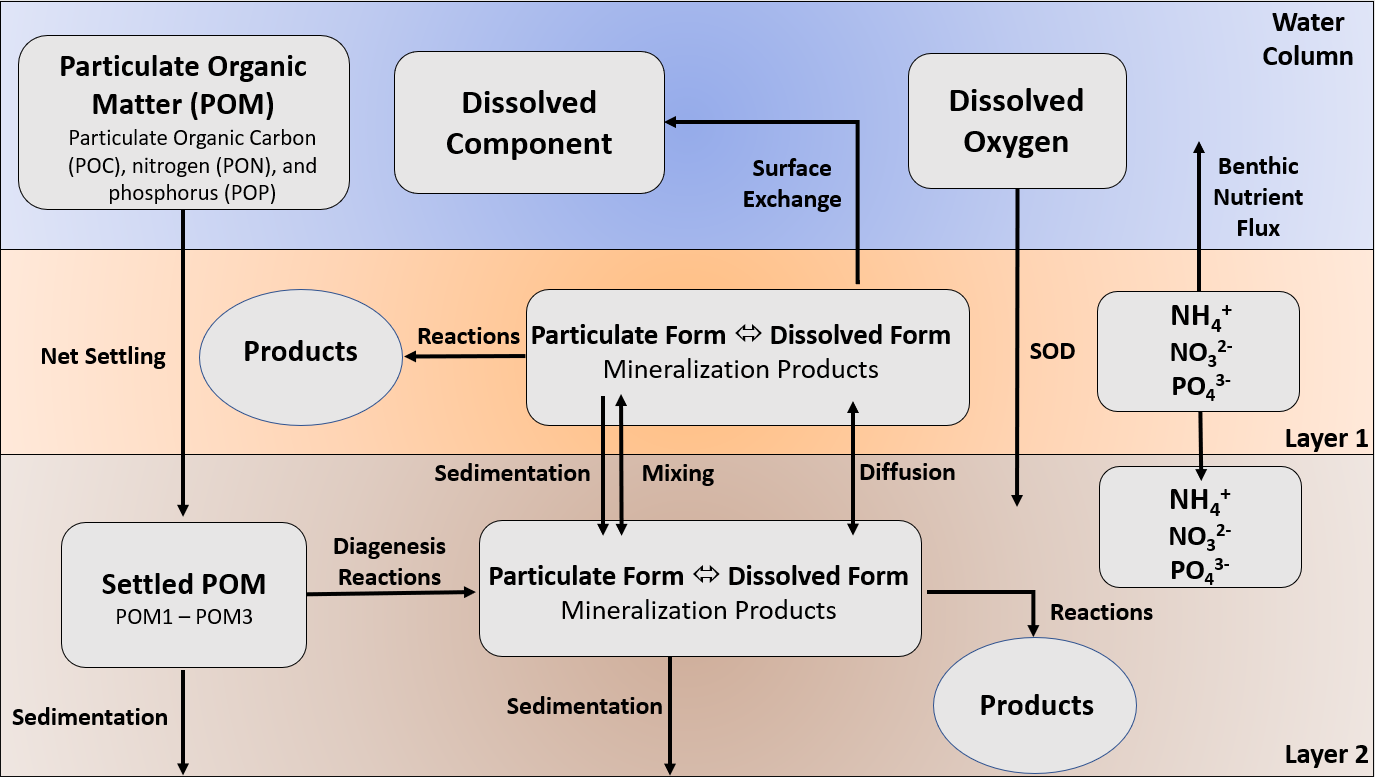
Figure S2. Flow chart illustrating the components and processes of WASP Sediment Diagenesis module.

# **Observed and Input Data**

## *3.1 Observed Data*

Table S2. Table providing data site locations of continuous sonde sampling with corresponding sampling year, month, location in water column, and collecting organization.

| **Data Site Location** | **Year** | **Months** | **Water Column** | **Organization** |
| --- | --- | --- | --- | --- |
| Little Narragansett Bay | 2019 | Jun-Oct | Surface | RIDEM |
| Pawcatuck Point | 2018 | Jun-Sep | Bottom | RIDEM |
|  | 2019 | Jun-Oct | Surface | RIDEM |
| Avondale Marina | 2019 | Jun-Oct | Both | RIDEM |
|  | 2020 | Jun-Oct | Both | RIDEM |
| Greenhaven Marina | 2018 | Jul-Oct | Both | USGS |
| Westerly Yacht Club | 2018 | Jun-Sep | Surface | RIDEM |
| Pawcatuck Rock | 2018 | Jun-Jul | Both | USGS |
|  | 2020 | Jan-Jul | Both | USGS |
| Viking Marina | 2018 | Jun-Sep | Surface | RIDEM |
|  | 2019 | Jun-Oct | Bottom | RIDEM |
|  | 2020 | Jun-Oct | Bottom | RIDEM |
| Route 1 | 2018 | Jun-Oct | Both | USGS |
|  | 2020 | Jan-Jul | Both | USGS |

Continuous (15-minute interval) sonde data and grab samples were collected. Additional sonde data were compiled from the National Water Information System Web (NEWISWeb) (U.S. Geographical Survey, 2023). Table S2 provides the data site, year(s), months, water column location, and collecting organization of continuous sonde data in the PRE. Refer to Figure 1 for the relative locations of each data site. The arrangement of sondes throughout the system allows for the assessment of a variety of water quality parameters with high spatial and temporal resolution. All continuous data can be found in Section 6. Files provided by RIDEM have continuous data on an interval of 15 minutes with sondes collecting water temperature (℃, column B), salinity (ppt, column C), dissolved oxygen (mg/L, column D), depth (m, column E), and chlorophyll *a* (µg/L, column F). Corresponding timestamps are in column A. Raw continuous data is found on the third page of each excel file titled by the location name. Additional descriptions of sonde placement and collected data can be found in Section 6 “SondeDataSummary” files.

USGS data are also found in Section 6 and are located on the sole Excel sheet within each file. USGS sondes have surface and bottom data simultaneously and record samples on 5-minute intervals. These sondes provide surface and bottom water temperature (℃, columns D and F), specific conductivity (µS/cm @ 25℃, columns H and J), dissolved oxygen (mg/L, columns L and N), and salinity (PSU, columns R and T). Chlorophyll *a* data from USGS were not used due to incompatibility of observed RFU units with µg/L WASP output. Corresponding timestamps for the observed data can be found in column C.

Grab samples were also used for nutrient evaluation. Grab samples were collected in the summer and fall of 2019 at various locations (Little Narragansett Bay, Pawcatuck Point, Pawcatuck Rock, and Viking Marina) approximately every two weeks when sondes were cleaned or replaced. Observed data used in the PRE model evaluation were salinity (ppt), water temperature (℃), chlorophyll *a* (µg/L), dissolved oxygen (mg/L), ammonium (mg/L as N), nitrate + nitrite (mg/L as N), orthophosphate (mg/L as P), total nitrogen (mg/L as N), and total phosphorus (mg/L as P). Nutrients were collected in (µg/L) and converted to fit WASP output. CBOD values would have been useful to this study, but nearly all samples were below the detection limit of 50 mg/L.

## *3.2 Input Data*

The PRE model has an upstream boundary (confluence of Green-Ashaway River and the Pawcatuck River) at Segment 1, an incoming tributary (Shunock River) at Segment 5, a summation of tributaries into Segment 17 (Little Narragansett Bay), and a downstream tidal boundary at Segment 17 (Fishers Island Sound). The upstream boundary is upstream of all tidal influence. Flow boundaries require inflow (m^3^/s) and concentrations provided by HSPF output (Lupo et al, 2021). HSPF also provided runoff and daily mass loads for each WASP segment (kg/d), as well as flow and associated concentrations from WWTPs (Segments 11 and 12). The tidal boundary required tidal height (m) and boundary concentrations. Data sources used in HSPF and WASP model development are provided in the Section 6 of the SI. Boundary concentrations include salinity (ppt), water temperature (℃), total suspended solids (TSS, mg/L), DO (mg/L), all nutrients (mg/L), and phytoplankton (mg chl *a*/L).

# **Parameterization**

Section 4 contains Tables S3 and S4 which provide the constant and parameter values. These determine aspects such as initial conditions, water body characteristics (heat exchange, division of solar radiation, settling rates, etc.), nutrient kinetics, and phytoplankton and macroalgae activity. Parameterization refers to the process of assigning values to constants while calibration refers to refining values to improve model function. Italicized values within Table S3 indicate those that were parameters of focus within calibration.

Initial parameterizations of the PRE model were based on modeling work in the Niantic River Estuary (Vaudrey et al, 2020), Narragansett Bay (Dettman, 2020), and suggested values from WASP documentation. Calibration was performed using visual analyses to capture trends and ranges of observed values. Model statistics, R-squared, Root Mean Square Error (RMSE), and Nash-Sutcliffe, were used to supplement calibration and assess model performance. As this study is not a best-fit exercise, statistics were not the primary method of model assessment. Values of calibrated model constants and parameters are listed in Tables S3 and S4 respectively. Details on HSPF model parameterization and calibration are provided in the Pawcatuck River Watershed Final HSPF Modeling Report in SI Section 6 (Lupo et al., 2022).

Table S3. Table of all WASP constants applied to model function. Table includes constant group, constant name, and associated value.

|  | **Constant** | **Value** |
| --- | --- | --- |
| **Global Constants** | Latitude- degrees | 41.3474 |
|  | Longitude- degrees | -71.8271 |
|  | Salinity Simulation Option (1 = Salinity- 2 = TDS) | 1 |
| **Water Temperature** | Heat exchange option (0=full heat balance- 1=equilibrium temperature) | 0 |
|  | Coefficient of bottom heat exchange- Watts m-2 °C-1 | 0.4 |
|  | Ice switch (0 = no ice solution- 1=ice solution- 2=detailed ice solution) | 0 |
| **Inorganic Nutrient Kinetics** | Nitrification Rate Constant @ 20℃ (1/day) | *0.1* |
|  | Denitrification Rate Constant @ 20℃ (1/day) | *0.09* |
|  | Nitrification Temperature Coefficient | 1.07 |
|  | Denitrification Temperature Coefficient | 1.04 |
|  | Minimum Temperature for Nitrification Reaction (degree C) | 4 |
|  | Half Saturation Constant for Nitrification Oxygen Limit (mg O2/L) | 2 |
|  | Half Saturation Constant for Denitrification Oxygen Limit (mg O2/L) | 0.1 |
| **Inorganic Nutrient Partitioning** | Ammonia Partition Coefficient to Water Column Solids TSS-S (L/kg) | 100 |
|  | Ammonia Partition Coefficient to Water Column Solids TSS-C (L/kg) | 100 |
|  | Orthophosphate Partition Coefficient to Water Column Solids TSS-S (L/kg) | 0 |
|  | Orthophosphate Partition Coefficient to Water Column Solids TSS-C (L/kg) | 0 |
| **Organic Nutrients** | Detritus Dissolution Rate (1/day) | 0.2 |
|  | Dissolved Organic Nitrogen Mineralization Rate Constant @ 20℃ (1/day) | 0.1 |
|  | Dissolved Organic Phosphorus Mineralization Rate Constant @ 20℃ (1/day) | 0.25 |
|  | Temperature Correction for detritus dissolution | 1.07 |
|  | Dissolved Organic Nitrogen Mineralization Temperature Coefficient | 1.07 |
|  | Dissolved Organic Phosphorus Mineralization Temperature Coefficient | 1.07 |
| **CBOD** | CBOD Decay Rate Constant @ 20℃ (1/day) | *0.3* |
|  | CBOD Decay Rate Temperature Correction Coefficient | 1.05 |
|  | CBOD Half Saturation Oxygen Limit (mg O2/L) | 1.05 |
|  | Fraction of Detritus Dissolution to CBOD | 0 |
|  | Fraction of CBOD Carbon Source for Denitrification | 1.05 |
| **Dissolved Oxygen** | Reaeration Option (0=Covar- 1= O’Connor- 2=Owens- 3=Churchill- 4=Tsivoglou, 5=Zappa (EFDC-Linkage) | 0 |
|  | Minimum Reaeration Velocity (m/day) | 0 |
|  | Maximum Allowable Calculate Reaeration Rate- per day | 24 |
|  | Waterbody Type Used for Wind Driven Reaeration Rate | 1 |
|  | Oxygen to Carbon Stoichiometric Ratio | 2.667 |
|  | Use Total Depth of Water Column for Reaeration | 0 |
| **Phytoplankton** | Phytoplankton Maximum Growth Rate Constant @@ 20℃ (1/day) | *4.5* |
|  | Phytoplankton Carbon to Chlorophyll Ratio (mg C/mg Chl) | *27.5* |
|  | Nitrogen fixation option (0 no- 1=yes) | 0 |
|  | Phytoplankton Respiration Rate Constant @ 20℃ (1/day) | *0.2* |
|  | Phytoplankton Death Rate Constant (Non-Zoo Predation) (1/day) | *0.075* |
|  | Phytoplankton Growth Temperature Coefficient | 1.07 |
|  | Optimal Temperature for Growth (C) | 22 |
|  | Shape parameter for below optimal temperatures | 0.01 |
|  | Shape parameter for above optimal temperatures | 0.001 |
|  | Phytoplankton Optimal Light Saturation as PAR (watts/m2) | 300 |
|  | Phytoplankton Respiration Temperature Coefficient | 1.045 |
|  | Phytoplankton Half-Sat. for Mineralization Rate (mg Phyt C/L) | 1.00E-06 |
|  | Phytoplankton Half-Saturation Constant for N Uptake (mg N/L) | *0*.*02* |
|  | Phytoplankton Half-Saturation Constant for P Uptake (mg P/L) | *0.008* |
|  | Phytoplankton Half-Saturation Constant for Si Uptake (mg Si/L) | 0.04 |
|  | Fraction of Phytoplankton Respiration Recycled to Organic N | 0.2 |
|  | Fraction of Phytoplankton Respiration Recycled to Organic P | 0.2 |
| **Light** | Light Option (0 - light from lat-long; 1 - input diel light; 2 - input daily light- calculated diel light) | 1 |
|  | Fraction of Light that is PAR (Photosynthetically Active Radiation) | 0.464 |
|  | Fraction of Light that is Infrared | 0.5 |
|  | Fraction of Light that is Ultraviolet | 0.036 |
|  | Fraction of solar radiation reflected at the water surface | 0.06 |
|  | Detritus & Solids Light Extinction Multiplier 1/m/(mg/L) | 0.05 |
|  | DOC Light Extinction Multiplier (Values Below Modify Global Value) | 0.2 |
| **Macroalgae** | Macro Algal Option: 1 = Floating forms (ave light) 2=Surface Algae (Top Light); 3 = submersed; 4 = benthic algae (not transported) | 1 |
|  | Macroalgae P:C Ratio (mg P/mg C) | 0.025 |
|  | Macroalgae Chl a:C Ratio (mg Chl/mg C) | 0.025 |
|  | Macroalgal Growth Model- 0 = Zero Order; 1 = First Order Macroalgal Growth Model- | 1 |
|  | Macroalgae Max Growth Rate (gD/m2-day- or 1/day) | *15* |
|  | 1 = use theta formulation; 2 = use optimal formulation | 2 |
|  | Optimal Temperature for Macroalgal Growth (℃) | *22* |
|  | Shape parameter for below optimal temperatures for Macroalgae | *0.02* |
|  | Shape parameter for above optimal temperatures for Macroalgae | *0.005* |
|  | Macroalgae Carrying Capacity for First Order Model (g D/m2) | 0 |
|  | Macroalgae Respiration Rate Constant (1/day) | *0.3* |
|  | Temperature Coefficient for Macro Algal Respiration | 1.09 |
|  | Internal Nutrient Excretion Rate Constant for Macroalgae (1/day) | 0.15 |
|  | Temperature Coefficient for Macro Algal Nutrient Excretion | 1.07 |
|  | Macroalgae Death Rate Constant (1/day) | *0.075* |
|  | Temperature Coefficient for Macro Algal Death | 1.07 |
|  | if = 0- not considered- if = 1 then salt-water optimal model- 2 fresh water | 1 |
|  | Optimal Salinity for marine Macroalgal Growth (ppt) | 18 |
|  | Shape parameter for below optimal salinity for Macroalgae | 0.005 |
|  | Shape parameter for above optimal salinity for Macroalgae | 0.005 |
|  | Macro Algal Half Saturation Uptake Constant for Extracellular Nitrogen (mg N/L) | *0.2359* |
|  | Macro Algal Half Saturation Uptake Constant for Extracellular Phosphorus (mg P/L | *0.0117* |
|  | Macro Algal Light Constant for growth (Langley’s/day) | 100 |
|  | Macroalgae ammonia preference (mg N/L) | 0.03 |
|  | Minimum Cell Quota of Internal Nitrogen for Macro Algal Growth (mgN/gDW) | 1 |
|  | Minimum Cell Quota of Internal Phosphorus for Macro Algal Growth (mgP/gDW) | 0.75 |
|  | Maximum Nitrogen Uptake Rate for Macroalgae (mgN/gDW-day) | *60.2645* |
|  | Maximum Phosphorus Uptake Rate for Macro Algae (mgP/gDW-day) | *4.185* |
|  | Half Saturation Uptake Constant for Macro Algal Intracellular Nitrogen (mgN/gDW) | 0.2 |
|  | Half Saturation Uptake Constant for Macro Algal Intracellular Phosphorus (mgP/gD | 0.156 |
|  | Grazing rate on Macroalgae | 0.13 |
|  | Macroalgae D:C Ratio (mg D/mg C) | *2.5* |
|  | Macroalgae N:C Ratio (mg N/mg C) | *0.18* |
|  | Macroalgae O2:C Production (mg O2/mg C) | *0.2* |
|  | Fraction of Macroalgae Recycled to Organic N | 0.3 |
|  | Fraction of Macroalgae Recycled to Organic P | 0.3 |
|  | Macroalgae Transport Drag Fraction (0 to 1) | 0 |
|  | Macro Algal Self shading coefficient (m3m-1 g-dw-1) | 0.01 |
| **Sediment Diagenesis**  **Sediment Diagenesis** | Activate Sediment Diagenesis Model (1=On- 0=Off) | 1 |
|  | 1=Read/Create Dynamic SOD Restart File (SOD.RST) Automatically Created | 1 |
|  | Determines if a steady-state calculation sets initial conditions (1=No-0=Yes) | 1 |
|  | Maximum error for testing convergence of the steady state solute | 0.001 |
|  | Maximum number of iterations of steady-state solution | 1000 |
|  | Salinity con. (ppt) for determining whether methane or Sulfide SOD | 1 |
|  | Determines whether fresh or saltwater nitrification/denitrification rates | 1 |
|  | Solid’s concentration in Layer 1 kg/L | 0.5 |
|  | Thickness of active sediment layer cm | 0.1 |
|  | Solid’s concentration in Layer 2 kg/L | 0.5 |
|  | Diffusion coefficient between layers 1 and 2 (m2/day) | 0.0025 |
|  | Temperature coefficient for Dd | 1.08 |
|  | Burial velocity for layer 2 to inactive sediments (m/day) | 6.85E-06 |
|  | Diffusion coefficient for particle mixing (m2/day) | 0.0006 |
|  | Temperature coefficient for Dp | 1.117 |
|  | Reference POC (O2 EQ. =0. 2.67) measurement for particle mixing | 0.2667 |
|  | Decay constant for benthic stress (1/day) | 0.02 |
|  | Particle mixing half-saturation constant for oxygen (gO2/m3) | 4 |
|  | Nitrogen Constants: Fraction PON to G1 | 0.65 |
|  | Diagenesis rate for PON G1 | 0.035 |
|  | Diagenesis rate for PON G2 | 0.0018 |
|  | Fraction PON to G2 | 0.25 |
|  | Temperature coefficient for diagenesis of PON G1 | 1.1 |
|  | Temperature coefficient for diagenesis of PON G2 | 1.15 |
|  | Diagenesis rate for PON G3 | 0 |
|  | Temperature coefficient for diagenesis of PON G3 | 1.17 |
|  | Freshwater nitrification reaction velocity (m/day) | 0.1313 |
|  | Saltwater nitrification reaction velocity (m/day) | 0.1313 |
|  | Temperature coefficient for nitrification | 1.123 |
|  | Half-saturation coefficient for ammonia in the nitrification reaction (mg/L) | 0.728 |
|  | Half-saturation coefficient for oxygen in the nitrification reaction (mg/L) | 0.37 |
|  | 2nd step reaction velocity for nitrification (NO2 to NO3) (m/day) | 100 |
|  | Temperature coefficient for 2nd step reaction velocity | 1.123 |
|  | Half-saturation coefficient for oxygen in the 2nd reaction step (mg O2/L) | 0.37 |
|  | Freshwater denitrification reaction velocity in layer 1(m/day) | 0.1 |
|  | Saltwater denitrification reaction velocity in layer 1 (m/day) | 0.1 |
|  | Temperature coefficient for denitrification | 1.08 |
|  | Denitrification reaction velocity in layer 2 (m/day) | 0.25 |
|  | Nitrogen partition coefficient (L/kg) | 1 |
|  | Phosphorus: Fraction POP to G1 | 0.65 |
|  | Diagenesis rate for POP G1 | 0.035 |
|  | Diagenesis rate for POP G2 | 0.0018 |
|  | Phosphorus: Fraction POP to G2 | 0.2 |
|  | Temperature coefficient for diagenesis of POP G1 | 1.1 |
|  | Temperature coefficient for diagenesis of POP G2 | 1.15 |
|  | Diagenesis rate for POP G3 | 0 |
|  | Temperature coefficient for diagenesis of POP G3 | 1.17 |
|  | Phosphorus partition coefficient in layer 2 (L/kg) | 20 |
|  | Incremental freshwater partition coefficient in layer 1 | 20 |
|  | Incremental saltwater partition coefficient in layer 1 | 20 |
|  | Critical oxygen concentration in layer 1 incremental phosphate sorption (mgO2/L) | 2 |
|  | Carbon Constants: Fraction CBODu to G1 | 0.65 |
|  | Diagenesis rate for CBODu G1 | 0.035 |
|  | Diagenesis rate for CBODu G2 | 0.0018 |
|  | Fraction CBODu to G2 | 0.2 |
|  | Temperature coefficient for diagenesis of CBODu G1 | 1.1 |
|  | Temperature coefficient for diagenesis of CBODu G2 | 1.15 |
|  | Diagenesis rate for CBODu G3 | 0 |
|  | Temperature coefficient for diagenesis of CBODu G3 | 1.17 |
|  | Methane oxidation reaction velocity (m/day) | 0.7 |
|  | Temperature coefficient for methane oxidation | 1.079 |
|  | Half-saturation coefficient for oxygen in oxidation of methane (mg/L) | 0.37 |
|  | Reaction velocity for dissolved sulfide oxidation in layer 1 (m/day) | 0.2 |
|  | Reaction velocity for particulate sulfide oxidation in layer 1 (m/day) | 0.4 |
|  | Temperature coefficient for sulfide oxidation | 1.079 |
|  | Sulfide oxidation normalization constant (mg/L) | 4 |
|  | Sulfide partition coefficient in layer 1 (L/kg) | 100 |
|  | Sulfide partition coefficient in layer 2 (L/kg) | 100 |
|  | Algae Constants: Fraction settled algae to G1 | 0.65 |
|  | Algae Constants: Fraction settled algae to G2 | 0.2 |
|  | Dissolution Rate of particulate biogenic silica at 20c (1/day) | 0.5 |
|  | Temperature Effect on Silica Dissolution | 1.1 |
|  | Silica Saturation Concentration in Porewater (mg si/m**3) | 40000 |
|  | Incremental Change (Mult) for freshwater in Partition Coeff. Si as DO | 10 |
|  | Partition Coefficient between Dissolved/Sorbed Silica in Layer 2 | 100 |
|  | Half Saturation Constant of Dissolved Silica in Dissolution Reaction | 5.00E+07 |
|  | Critical Oxygen Concentration for Silica Sorption | 1 |
| **Solids Transport** | Solids option. 0: input vs- vd- vr; 1: vs- vd- vr calculated from shear stress TSS-S | 0 |
|  | Solids option. 0: input vs- vd- vr; 1: vs- vd- vr calculated from shear stress TSS-C | 0 |
|  | Particle diameter for Solid- mm TSS-S | 0.25 |
|  | Particle diameter for Solid- mm TSS-C | 0.033 |

Table S4. Table of all WASP parameters used in model function. Includes parameter group name, parameter name, applicable WASP system, and value. Parameter values remain constant for each segment in the PRE model. *Denotes one parameter where this was not an option: Segments 1-5 = 1, Segments 6-9 = 2, Segments 10-16 = 3, Segment 17 = 4.

| **Parameter Group** | **Parameter** | **System** | **Value** |
| --- | --- | --- | --- |
| Settling Rates | Solid Settling Velocity in Segment [m/day] | Silt | 1 |
|  | Solid Settling Velocity in Segment [m/day] | Clay | 0.15 |
|  | Solid Resuspension Velocity in Segment [m/day] | Silt | 0.0001 |
|  | Solid Resuspension Velocity in Segment [m/day] | Clay | 0.0001 |
|  | Phytoplankton Settling Velocity in Segment [m/day] | Phytoplankton 1 | 0.05 |
|  | Settling Velocity of Segment Particulate Organic Matter [m/day] | Particulate Organic Matter 1 | 0.05 |
| Sediment Diagenesis | Sediment Diagenesis Segment Attached to this Segment | Dissolved Oxygen 1 | 1-4* |
|  | PON Initial Condition for Sediment Diagenesis Segment | Dissolved Oxygen 1 | 2 |
|  | POP Initial Condition for Sediment Diagenesis Segment | Dissolved Oxygen 1 | 0.5 |
|  | POC Initial Condition for Sediment Diagenesis Segment | Dissolved Oxygen 1 | 20 |
|  | POSi Initial Condition for Sediment Diagenesis Segment | Dissolved Oxygen 1 | 1 |
|  | Fraction of [PO-N/P/C] in Class 1 | Dissolved Oxygen 1 | 0.05 |
|  | Fraction of [PO-N/P/C] in Class 2 | Dissolved Oxygen 1 | 0.08 |

# **5. Calibration and Model Evaluation**

## *5.1 Hydrodynamics*

Section 5.1 contains Tables S5 and S6 which provide supplemental information to Figure 2. Table S5 contains the simulated and observed ranges and means of all continuous salinity and water temperature while Table S6 provides statistics of R-squared, root mean square error (RMSE), and Nash-Sutcliffe. Throughout Section 5 “statistics” will always refer to those previously listed. Additional Figures S3 and S4 are scatterplot graphs showing the simulated versus observed data for salinity and water temperature.

Table S5. All simulated and observed means and ranges of data presented in Figure 2. Simulated means and ranges are presented from same time as corresponding observed data. *( ) Denotes ranges and means of simulated values for the full model simulation.

| **Segment** | **Year** | **Salinity (ppt)** | | **Water Temp (℃)** | |
| --- | --- | --- | --- | --- | --- |
|  |  | Range | Average | Range | Average |
| Segment 17 | 2019 | 19.9-31.9 (14.2-33.1)* | 28.7 (28.0)* | 15.4-26.9 (0-26.9) | 21.2 (11.4) |
| Little Narragansett Bay |  | 19.3-32.4 | 29.2 | 16.9-26.5 | 21.2 |
| Segment 16 | 2018 | 16.8-31.9 (0.13-32.4) | 27.5 (20.5) | 17.0-26.6 (0-26.9) | 22.3 (11.7) |
| Pawcatuck Point Bottom |  | 24.5-31.6 | 29.9 | 17.6-26.7 | 22.6 |
| Segment 16 | 2019 | 3.26-29.8 | 22.8 | 13.4-26.9 | 21 |
| Pawcatuck Point Surface |  | 0.100-31.3 | 26.8 | 15.4-26.7 | 21.7 |
| Segment 14 | 2018 | 2.54-29.4 (0-30.6) | 21.0 (11.7) | 16.9-26.1 (0-27.6) | 22.3 (12.0) |
| Greenhaven Surface |  | 2.90-30.0 | 20.5 | 17.0-29.5 | 24.1 |
| Greenhaven Bottom |  | 9.80-34.0 | 29.4 | 19.0-27.6 | 23.9 |
| Segment 14 | 2019 | 0.145-27.0 | 14.3 | 10.4-27.2 | 20.2 |
| Avondale Surface |  | 3.84-31.1 | 24.8 | 12.4-26.4 | 20.7 |
| Avondale Bottom |  | 16.9-31.4 | 28.9 | 13.2-23.9 | 19.9 |
| Segment 14 | 2020 | 10.6-26.6 | 20.2 | 19.6-25.8 | 23.8 |
| Avondale Surface |  | 11.4-30.7 | 25.9 | 18.2-28.0 | 23.1 |
| Avondale Bottom |  | 26.4-31.5 | 30.1 | 17.6-25.6 | 21.4 |
| Segment 12 | 2018 | 2.46-24.33 (0-27.1) | 15.2 (6.11) | 16.7-28.0 (0-28.0) | 23.1 (12.3) |
| Westerly Yacht Club |  | 2.20-29.9 | 19.4 | 19.0-29.7 | 24.6 |
| Segment 12 | 2018 | 8.57-22.6 | 14.2 | 21.4-28.0 | 24.3 |
| Pawcatuck Rock Surface |  | 1.80-23.0 | 10.8 | 20.9-29.9 | 25.4 |
| Pawcatuck Rock Bottom |  | 8.30-30.0 | 23.8 | 19.9-27.1 | 23.2 |
| Segment 12 | 2020 | 0.046-15.6 | 3.75 | 0-25.6 | 10.7 |
| Pawcatuck Rock Surface |  | 0.200-30.0 | 4.19 | 0.3-27.0 | 11.6 |
| Pawcatuck Rock Bottom |  | 0.200-35.0 | 17.6 | 1.40-25.4 | 10.7 |
| Segment 10 | 2018 | 0.002-17.6 (0-22.2) | 6.14 (2.15) | 16.8-27.5 (0-28.1) | 23.2 (12.6) |
| Viking Marina Surface |  | 0.18-28.5 | 15.7 | 18.1-28.9 | 24.3 |
| Segment 10 | 2019 | 0-12.5 | 1.81 | 10.9-26.7 | 20 |
| Viking Marina Bottom |  | 0.06-29.7 | 23.7 | 12.5-24.6 | 20.8 |
| Segment 10 | 2020 | 0.311-14.9 | 5.07 | 19.6-26.9 | 24.3 |
| Viking Marina Bottom |  | 2.45-27.8 | 23.2 | 18.1-27.0 | 23.2 |
| Segment 9 | 2018 | 0-14.5 (0-20.0) | 2.86 (1.13) | 16.9-27.4 (0-28.3) | 22.8 (12.6) |
| Route 1 Surface |  | 0-29 | 16.8 | 16.3-28.8 | 23.7 |
| Route 1 Bottom |  | 0-29 | 25.3 | 16.3-26.4 | 22.9 |
| Segment 9 | 2020 | 0-4.24 | 0.137 | 0-26.0 | 11.4 |
| Route 1 Surface |  | 0-28 | 1.36 | 0-26.3 | 11.3 |
| Route 1 Bottom |  | 0-28 | 6 | 0-21.8 | 10.9 |

Tale S6. Table of statistics for hydrodynamic data presented in Figure 2 and Table S6 including R-squared, Root Mean Square Error (RMSE), and Nash-Sutcliffe.

| **Segment** | **Year** | **Salinity (ppt)** | | | **Water Temp (℃)** | | |
| --- | --- | --- | --- | --- | --- | --- | --- |
|  |  | R-Squared | RMSE | Nash-Sutcliffe | R-Squared | RMSE | Nash-Sutcliffe |
| Little Narragansett Bay | 2019 | 0.61 | 2.05 | -1.11 | 0.41 | 1.89 | -1.12 |
| Pawcatuck Point Bottom | 2018 | 0.12 | 2.99 | -14.5 | 0.33 | 1.78 | 0.14 |
| Pawcatuck Point Surface | 2019 | 0.29 | 6.25 | -7 | 0.58 | 1.97 | -0.34 |
| Greenhaven Surface | 2018 | 0.71 | 3.05 | 0.67 | 0.9 | 1.94 | 0.47 |
| Greenhaven Bottom |  | 0.25 | 9.23 | -25.7 | 0.59 | 1.25 | 0.24 |
| Avondale Surface | 2019 | 0.26 | 11.9 | -5.47 | 0.78 | 2.21 | 0.37 |
| Avondale Bottom |  | 0.014 | 15.9 | -199.7 | 0.67 | 2.67 | -0.37 |
| Avondale Surface | 2020 | 0.29 | 6.29 | -3.12 | 0.39 | 1.62 | 0.23 |
| Avondale Bottom |  | 0.042 | 10.2 | -166.2 | 0.37 | 2.72 | -1.43 |
| Westerly Yacht Club | 2018 | 0.32 | 5.94 | -0.33 | 0.68 | 1.95 | 0.18 |
| Pawcatuck Rock Surface | 2018 | 0.65 | 3.89 | 0.26 | 0.65 | 1.58 | 0.14 |
| Pawcatuck Rock Bottom |  | 0.001 | 11.3 | -11.9 | 0 | 2.5 | -1.86 |
| Pawcatuck Rock Surface | 2020 | 0.55 | 2.65 | 0.5 | 0.99 | 1.16 | 0.97 |
| Pawcatuck Rock Bottom |  | 0.23 | 16.6 | -1.48 | 0.95 | 2.12 | 0.85 |
| Viking Marina Surface | 2018 | 0.098 | 12.4 | -1.21 | 0.49 | 2.06 | -0.081 |
| Viking Marina Bottom | 2019 | 0.088 | 22.7 | -11.4 | 0.69 | 2.47 | -0.051 |
| Viking Marina Bottom | 2020 | 0.001 | 18.7 | -23.6 | 0.4 | 1.91 | -0.026 |
| Route 1 Surface | 2018 | 0.14 | 17 | -1.59 | 0.49 | 2.14 | 0.11 |
| Route 1 Bottom |  | 0.056 | 23.4 | -11.3 | 0.18 | 2.67 | -0.65 |
| Route 1 Surface | 2020 | 0.62 | 4.4 | 0.07 | 0.99 | 0.85 | 0.98 |
| Route 1 Bottom |  | 0.27 | 11.5 | -0.288 | 0.97 | 1.67 | 0.92 |


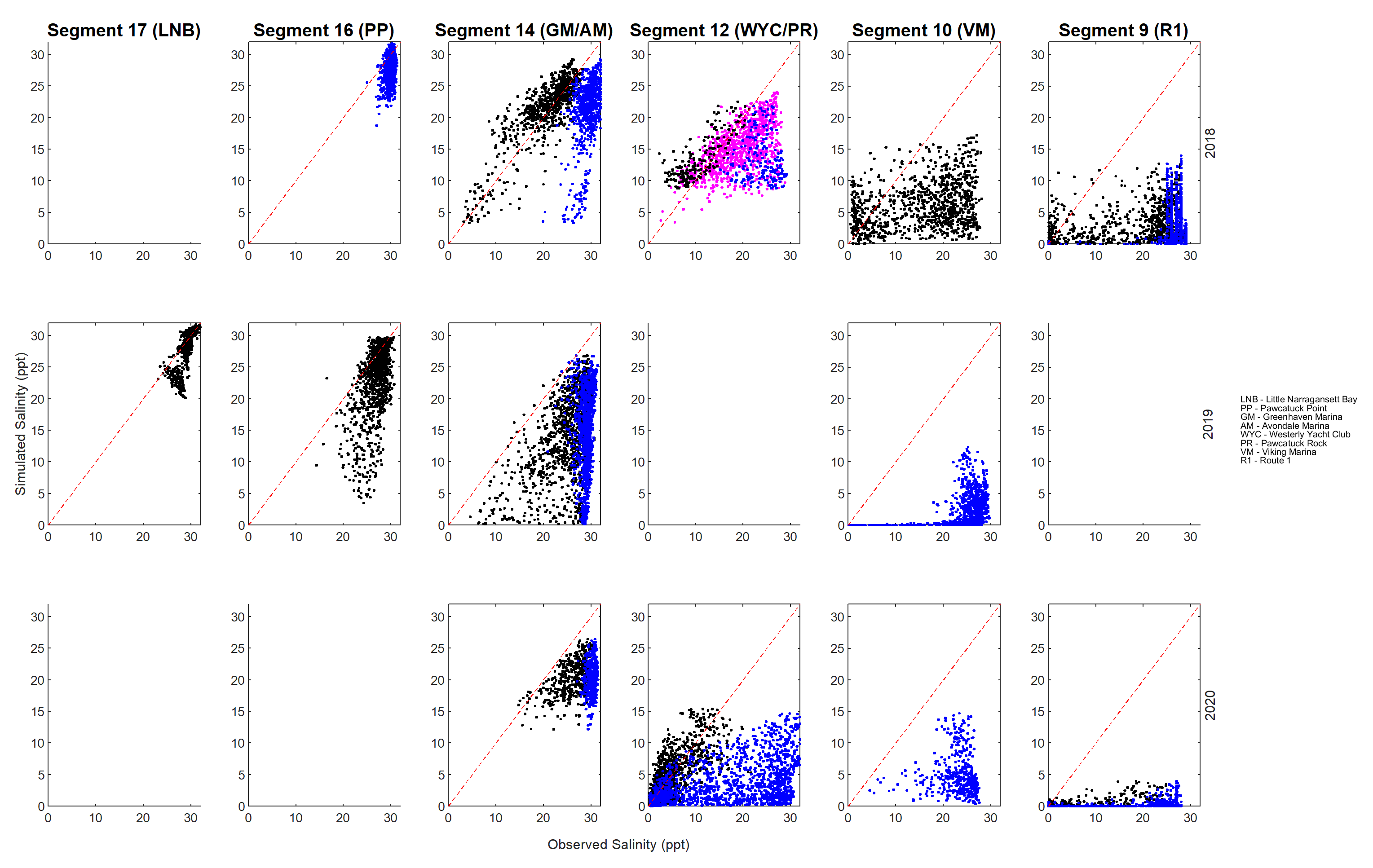
 Figure S3. Subplot containing salinity regressions supplementing information in Table S5 and S6. This figure contains simulated salinity versus observed surface salinity (black) and observed bottom salinity (blue). Magenta represents simulated salinity versus observed salinity at Westerly Yacht Club. Locations move upstream within the figure from left to right. Rows 1, 2, and 3 present data from 2018, 2019, and 2020 respectively. Site abbreviations are written to the right. Blank subplots are areas and times where no observed data was collected.


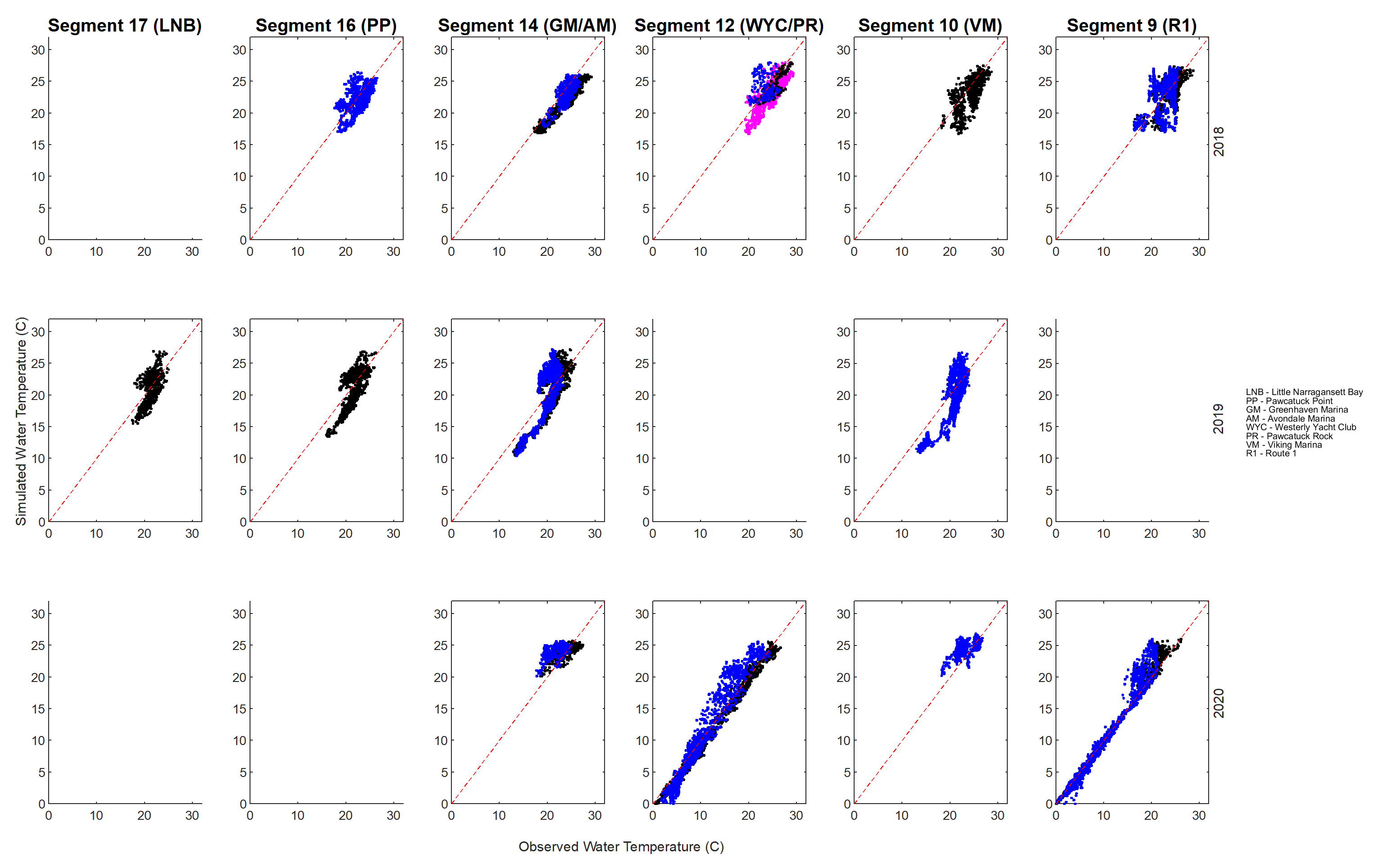


Figure S4. Subplot containing water temperature regressions supplementing information in Table S5 and S6. This figure contains simulated water temperature versus observed surface water temperature (black) and observed bottom water temperature (blue). Magenta represents simulated water temperature versus observed water temperature at Westerly Yacht Club. Locations move upstream within the figure from left to right. Rows 1, 2, and 3 present data from 2018, 2019, and 2020 respectively. Site abbreviations are written to the right. Blank subplots are areas and times where no observed data was collected.

## *5.2 Dissolved Oxygen and Phytoplankton*

Table S7 and Figure S5 provide additional information on simulated and observed dissolved oxygen (DO) presented in Figure 4. Table S7 contains all simulated and observed ranges and means of data in Figure 4, as well as statistics for these comparisons. Figure S5 provides scatter plots of simulated versus observed dissolved oxygen for all available data.

Table S7. Table of ranges, means, and statistics for simulated and observed dissolved oxygen data. This table presents data from all sondes in the PRE. Ranges and means shown in parentheses represent the simulated data at the same time of the observed.

| **Data Site** | **Year** | **Dissolved Oxygen (mg/L)** | | | | |
| --- | --- | --- | --- | --- | --- | --- |
|  |  | Range | Mean | R-Squared | RMSE | Nash-Sutcliffe |
| Little Narragansett Bay | 2019 | 1.9-17.0 (10.9-26.9) | 8.32 (14.4) | 0.02 | 7.4 | -19.55 |
| Pawcatuck Point Bottom | 2018 | 0.13-20.5 (4.33-20.7) | 7.25 (10.3) | 0.01 | 5.5 | -2.41 |
| Pawcatuck Point Surface | 2019 | 0.7-17.6 (6.73-15.7) | 8.22 (10.68) | 0.03 | 3.26 | -3.91 |
| Greenhaven Surface | 2018 | 4.00-14.3 (2.64-13.3) | 7.97 (6.72) | 0.13 | 3.37 | -4.97 |
| Greenhaven Bottom |  | 0-12.0 | 6.36 | 0.01 | 2.96 | -2.55 |
| Avondale Surface | 2019 | 2.20-12.3 (5.33-14.3) | 7.15 (8.06) | 0.07 | 1.94 | -1.03 |
| Avondale Bottom |  | 1.51-10.3 | 6.53 | 0.11 | 2.2 | -2.02 |
| Avondale Surface | 2020 | 3.58-13.8 (3.86-8.05) | 7.71 (6.08) | 0.21 | 2.08 | -1.17 |
| Avondale Bottom |  | 3.08-10.6 | 6.81 | 0.47 | 1.17 | 0.11 |
| Westerly Yacht Club | 2018 | 0.29-17.0 (2.85-7.53) | 7.48 (4.78) | 0 | 3.87 | -1.48 |
| Pawcatuck Rock Surface | 2018 | 5.70-14.4 (3.09-6.40) | 9.92 (4.59) | 0.02 | 5.62 | -15.2 |
| Pawcatuck Rock Bottom |  | 1.90-14.0 | 6.79 | 0.12 | 2.82 | -1.38 |
| Pawcatuck Rock Surface | 2020 | 3.70-16.5 (5.11-12.6) | 10.6 (9.57) | 0.72 | 1.48 | 0.28 |
| Pawcatuck Rock Bottom |  | 2.10-16.8 | 10.1 | 0.74 | 1.2 | 0.64 |
| Viking Marina Surface | 2018 | 0.02-14.1 (4.75-8.05) | 4.75 (6.27) | 0.01 | 2.99 | -0.37 |
| Viking Marina Bottom | 2019 | 0.20-8.69 (6.58-10.1) | 3.55 (7.56) | 0.09 | 4.36 | -4.81 |
| Viking Marina Bottom | 2020 | 0.04-9.77 (4.74-7.76) | 3.11 (6.50) | 0.17 | 3.67 | -4.72 |
| Route 1 Surface | 2018 | 0-10.9 (5.49-8.31) | 3.73 (7.06) | 0.12 | 4.5 | -1.01 |
| Route 1 Bottom |  | 0-9.30 | 1.9 | 0.29 | 6.3 | -4.2 |
| Route 1 Surface | 2020 | 0.80-14.1 (6.55-12.8) | 10.6 (9.74) | 0.76 | 1.44 | 0.62 |
| Route 1 Bottom |  | 0.50-14.0 | 9.85 | 0.7 | 2.14 | 0.59 |


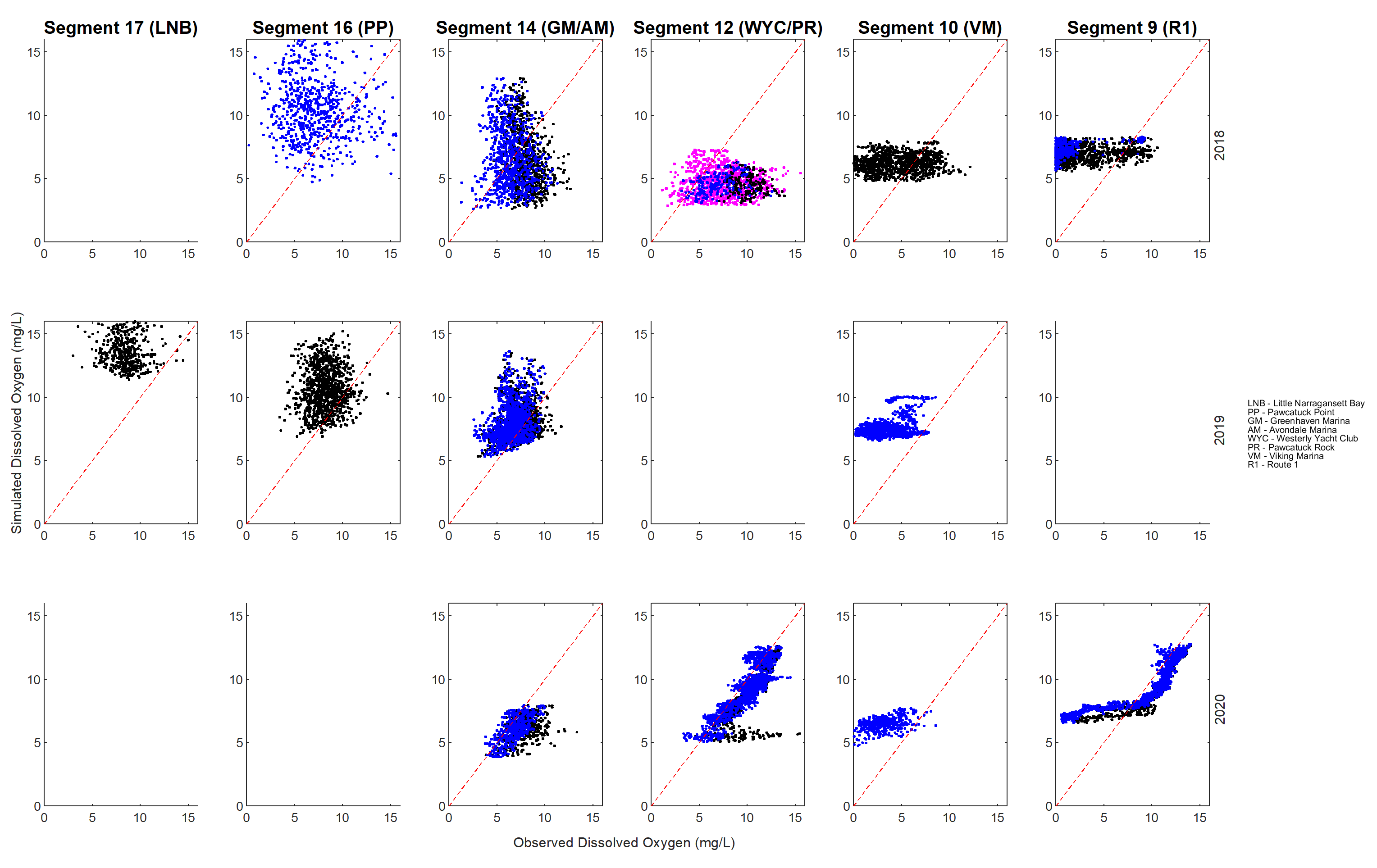


Figure S5. Subplot containing dissolved oxygen regressions supplementing information in Table S7. This figure contains simulated dissolved oxygen versus observed surface dissolved oxygen (black) and observed bottom dissolved oxygen (blue). Magenta represents simulated dissolved oxygen versus observed dissolved oxygen at Westerly Yacht Club. Locations move upstream within the figure from left to right. Rows 1, 2, and 3 present data from 2018, 2019, and 2020 respectively. Site abbreviations are written to the right. Blank subplots are areas and times where no observed data was collected.

Figure S6 presents the simulated loss of DO due to various processes throughout the PRE. WASP provides flux outputs which calculate the loss of DO due to CBOD oxidation (black, CBOD), SOD (SOD, magenta), and phytoplankton respiration (PHYTO, green). Outputs from the full model simulation are provided and move from downstream to upstream down the figure. Simulations in Figure S6 suggest that CBOD may be the largest driver of DO loss in the PRE. Simulated mean DO loss due to CBOD peaked in Segment 16 (-1.35 g/m^2^) and decreased moving upstream to -0.69 g/m^2^. DO loss due to CBOD was slightly lower in Segment 17 (-1.28 g/m^2^) and this may be the DO-sag represented by the Streeter-Phelps equation. Mean DO loss due to phytoplankton respiration generally increased moving upstream but decreased from Segment 12 to Segment 10 (-0.070 g/m^2^ to -0.048 g/m^2^). Mean DO loss due to SOD is lowest in Segments 16 and 14 (-0.17 g/m^2^ and -0.20 g/m^2^) and is highest in Segments 17 and 10 (-0.37 g/m^2^ to -0.32 g/m^2^).


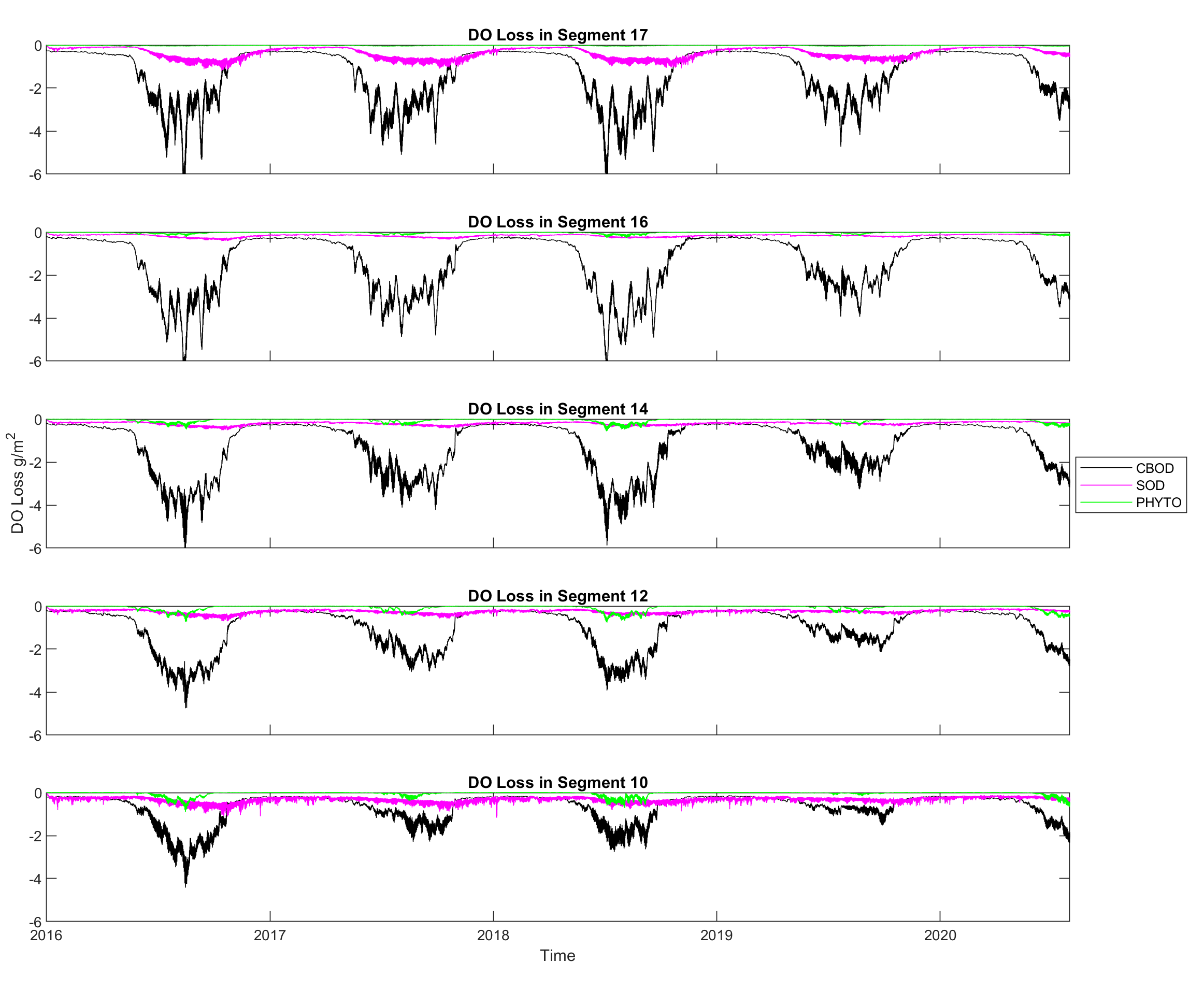


Figure S6. Simulated values of DO loss due to CBOD oxidation (black), SOD (magenta), and phytoplankton respiration (green) presented in various segments of the WASP model. Results are presented for the full model simulation.

Table S8 provides all simulated and observed ranges, means, and statistics of all phytoplankton (chl *a* µg/L) data. These data are also presented in Figure 4 of the main body. Figure S7 presents a scatter plot graph of simulated versus observed data for all data presented in Table S8.

Table S8. Table of ranges, means, and statistics for simulated and observed phytoplankton data. This table presents data from all sondes sampling chlorophyll *a* in µg/L. Ranges and means shown in parentheses represent the simulated data at the same time of the observed.

| **Segment** | **Year** | **Phytoplankton (chl *a* µg/L)** | | | | |
| --- | --- | --- | --- | --- | --- | --- |
|  |  | Range | Mean | R-Squared | RMSE | Nash-Sutcliffe |
| Little Narragansett Bay | 2019 | 0.96-34.2 (2.00-4.51) | 6.0 (2.95) | 0.02 | 4.04 | -1.17 |
| Pawcatuck Point Bottom | 2018 | 1.03-27.2 (1.75-14.7) | 5.65 (5.80) | 0.13 | 4.66 | -1.16 |
| Pawcatuck Point Surface | 2019 | 0-212.7 (1.26-11.0) | 17.9 (4.17) | 0.02 | 23.73 | -0.47 |
| Avondale Surface | 2019 | 2.54-83.0 (0.29-17.6) | 14.4 (5.77) | 0.07 | 11.27 | -1.22 |
| Avondale Bottom |  | 1.65-99.8 | 9.89 | 0.003 | 10.03 | -0.42 |
| Avondale Surface | 2020 | 2.28-71.0 (8.27-22.3) | 14.0 (13.9) | 0.44 | 6.6 | 0.36 |
| Avondale Bottom |  | 0.61-39.1 | 7.72 | 0.03 | 8.24 | -1.57 |
| Westerly Yacht Club | 2018 | 3.9-459.4 (0.64-36.5) | 30.0 (18.8) | 0.11 | 24.51 | -0.11 |
| Viking Marina Surface | 2018 | 0.1-448.5 (0.063-36.5) | 18.2 (14.0) | 0.05 | 18.76 | -0.05 |
| Viking Marina Bottom | 2019 | 1.30-136.0 (0-11.0) | 9.32 (2.04) | 0.001 | 11.57 | -0.69 |
| Viking Marina Bottom | 2020 | 1.65-118.1 (2.50-34.9) | 15.2 (14.5) | 0.19 | 11.38 | 0.09 |


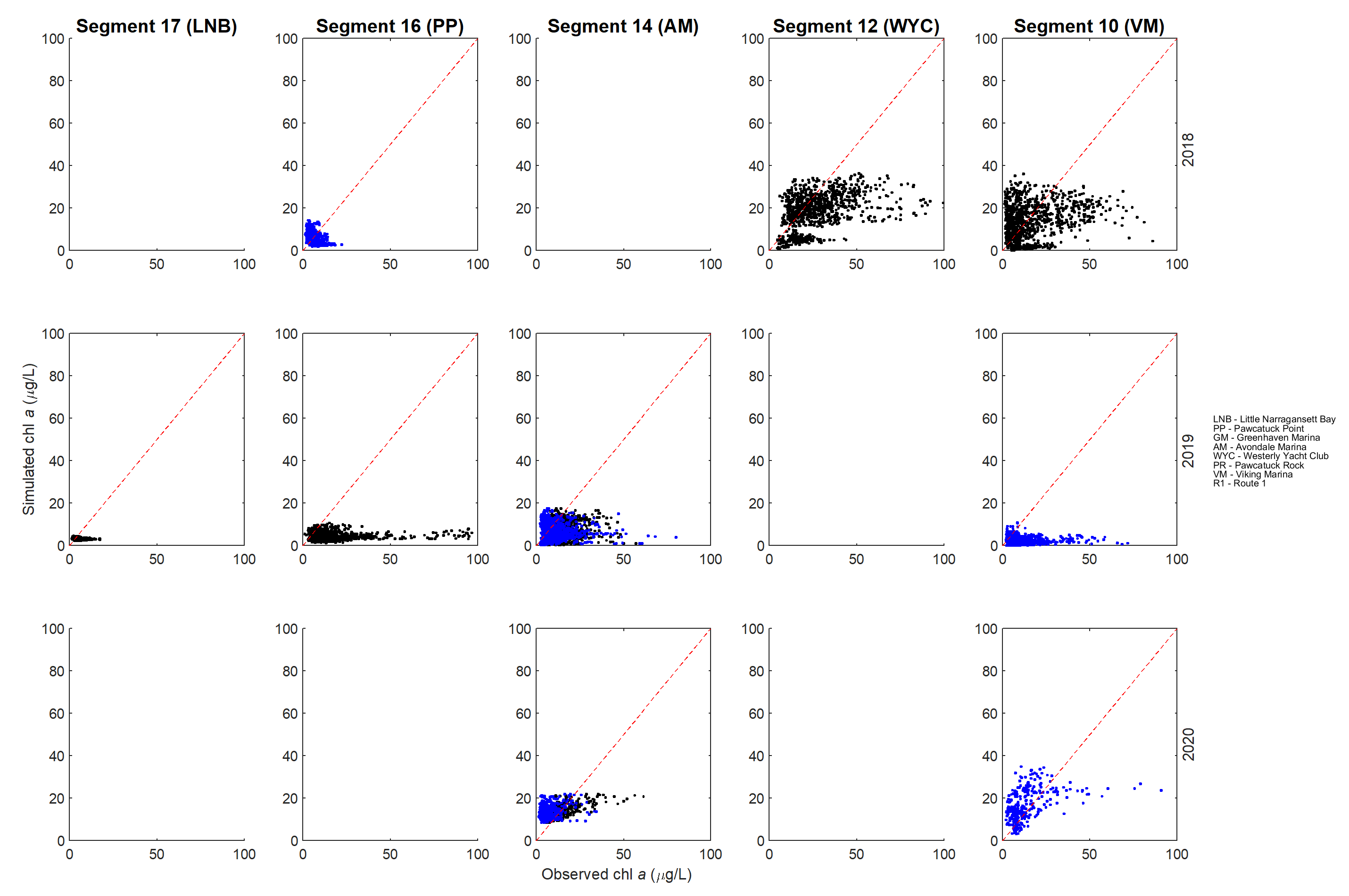


Figure S7. Subplot containing phytoplankton regressions supplementing information in Table S8. This figure contains simulated phytoplankton versus observed surface phytoplankton (black) and observed bottom phytoplankton (blue). Locations move upstream within the figure from left to right. Rows 1, 2, and 3 present data from 2018, 2019, and 2020 respectively. Site abbreviations are written to the right. Blank subplots are areas and times where no observed data was collected. Refer to Table S9 for the full ranges of observed data. The axis of each figure may not represent the maximum observed range to maintain figure resolution.

Figure S8 presents the simulated phytoplankton growth limitation factors via nitrogen (black) and phosphorus (red) for the full model simulation across multiple segments. WASP internally calculates various limitation factors on a scale of 0-1, where 0 is complete limitation and 1 is no limitation. Simulations presented in Figure S8 suggest that the PRE is largely a phosphorus limited system, where nitrogen limitation is similarly as severe only in Segment 17 (LNB). The mean simulated limitations for nitrogen and phosphorus respectively are 0.56 and 0.46 (Segment 17), 0.85 and 0.64 (Segment 16), 0.93 and 0.70 (Segment 14), 0.95 and 0.71 (Segment 12), and 0.95 and 0.66 (Segment 10). Often, north temperate estuaries are observed to be nitrogen limited, but some systems can be phosphorus limited for a variety of reasons. The N:P loading, loss rates of N and P via processes such as denitrification, nitrification, and residence time have all been observed to have a substantial impact on the relative abundance of available nitrogen and phosphorus (Forqurean et al., 1992).


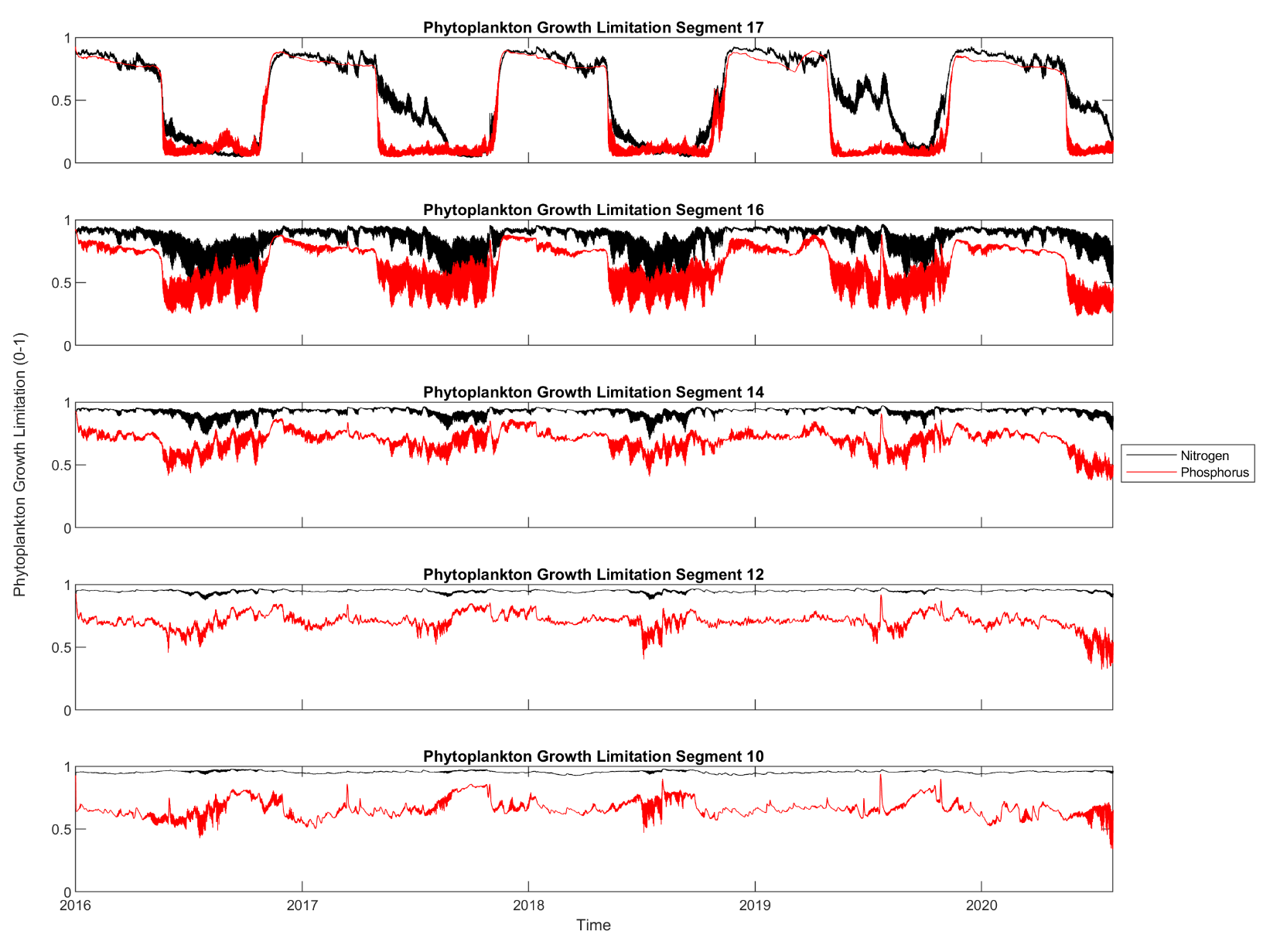


Table S8. Subplot containing simulated phytoplankton growth limitation factors. Segments move upstream from the top to bottom of the figure. Figure contains the nitrogen limitation factor (black) and the phosphorus limiting factor (red).

## *5.3 Nutrients*

Figure S9 presents simulated and observed nutrient (TN, TP, CBOD) and phytoplankton (chl *a*) concentrations. Statistics and scatterplot graphs were not presented for nutrient species due to the limited number of grab samples. Observed CBOD was limited as only two samples were below the detection limit of 50 mg/L. Observed [TN] decreases moving downstream and is generally higher in the surface waters than that of the bottom waters. This vertical gradient is not observed in LNB, suggesting the water column is homogenous in [TN]. No data is available to discern further spatial variability across the Bay. Observed [TP] values do not decrease moving downstream and are very low throughout the system. There is little to no difference between observed [TP] collected at different locations or vertically through the water column. The model captures the trends observed in both TN and TP for the summer of 2019. Simulated [TN] decreases moving downstream and is particularly low in Segment 17 (LNB) with a high of 0.32 mg/L. Simulated [TN] predicts seasonal variation in TN throughout the year with higher [TN] in the summer and fall and lower in winter months. That trend is clearer in the upstream segments as summer peaks are less distinctive downstream. The model also predicts low [TP] throughout the system. Simulated [TP] also suggests seasonal shifts with values higher in the summer and fall while the winter shows a decrease in mean concentration. Simulated [CBOD] suggest increasing concentrations moving downstream, unlike the trends observed for TN, TP, and phytoplankton. [CBOD] peaks become consistently higher moving downstream, occurring in the summer. Simulations suggest more variability in [CBOD] in warmer months than in those of the winter and spring, but interpretation is limited due to lack of observations.


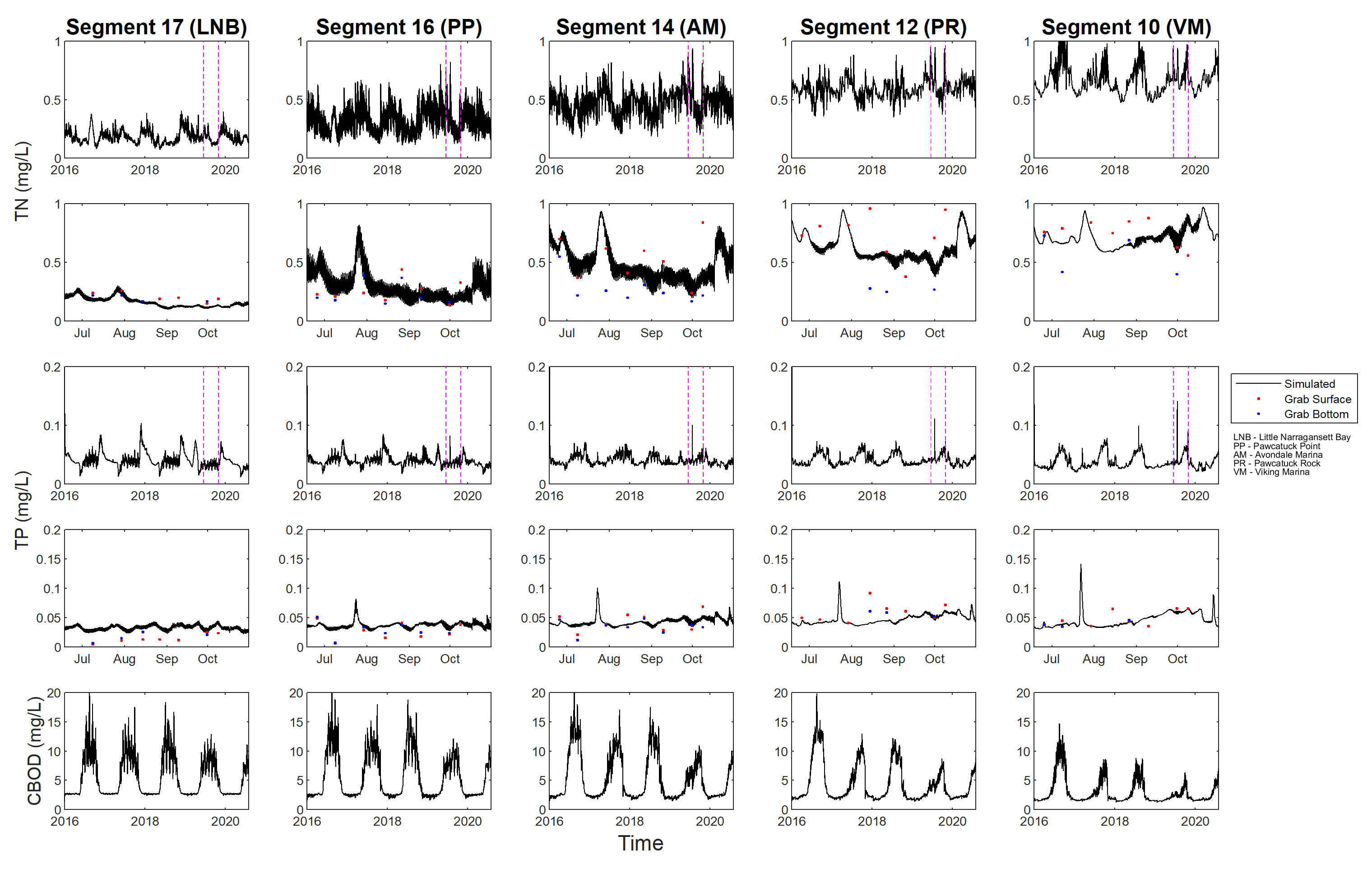


Figure S9. Simulated and observed total nitrogen (TN), total phosphorus (TP), and carbonaceous biological oxygen demand (CBOD). Black lines indicate simulated values, red dots are observed surface grab samples, and blue dots are observed bottom grab samples. Magenta lines show the focus period with observed data in 2019.

## *5.4 Spatial Variation – Heat Maps*


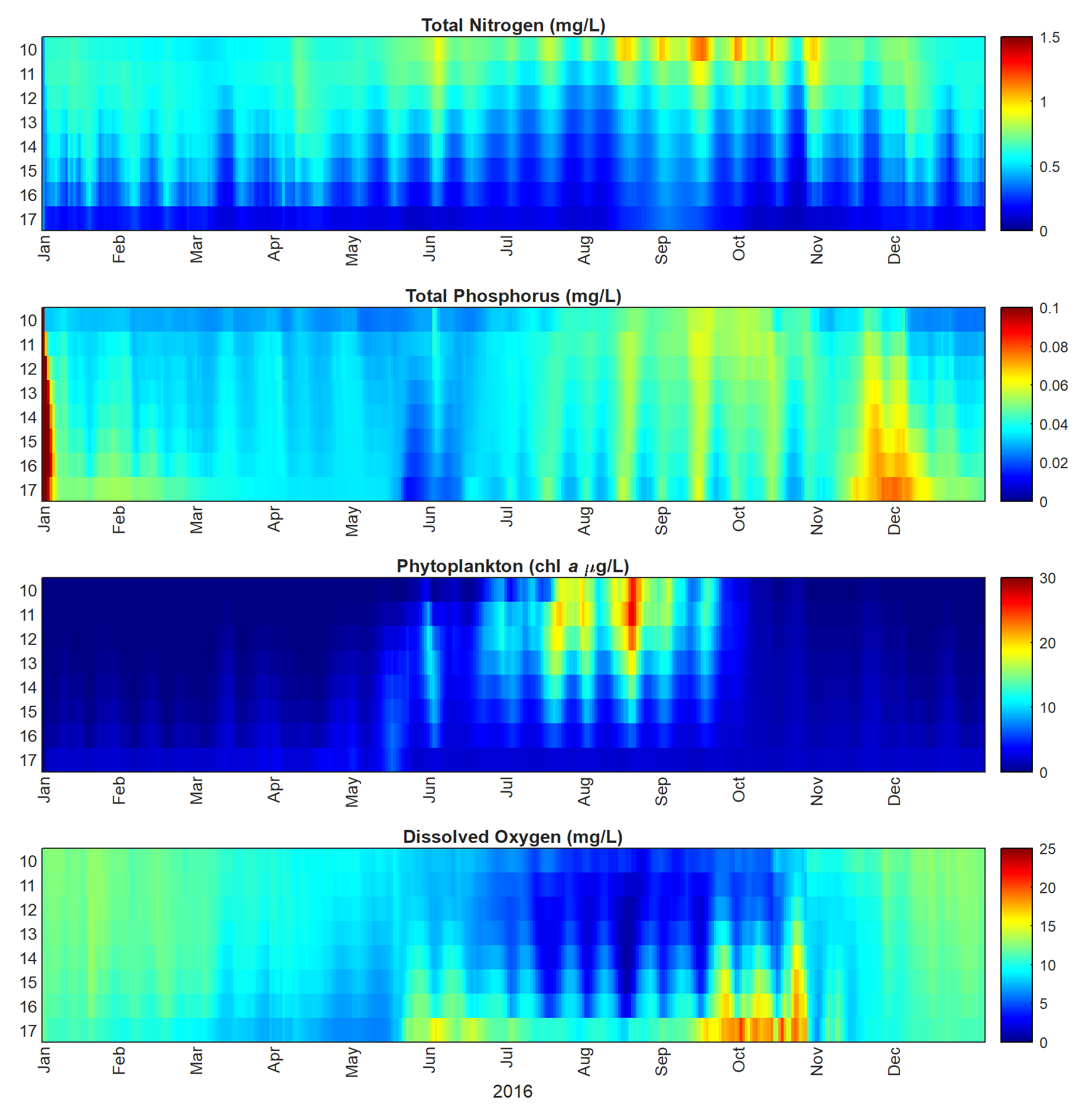


Figure S10. Heatmap of simulated concentrations of total nitrogen, total phosphorus, phytoplankton, and dissolved oxygen in the year of 2016. Data are presented for WASP segments 10-17 (y-axis).


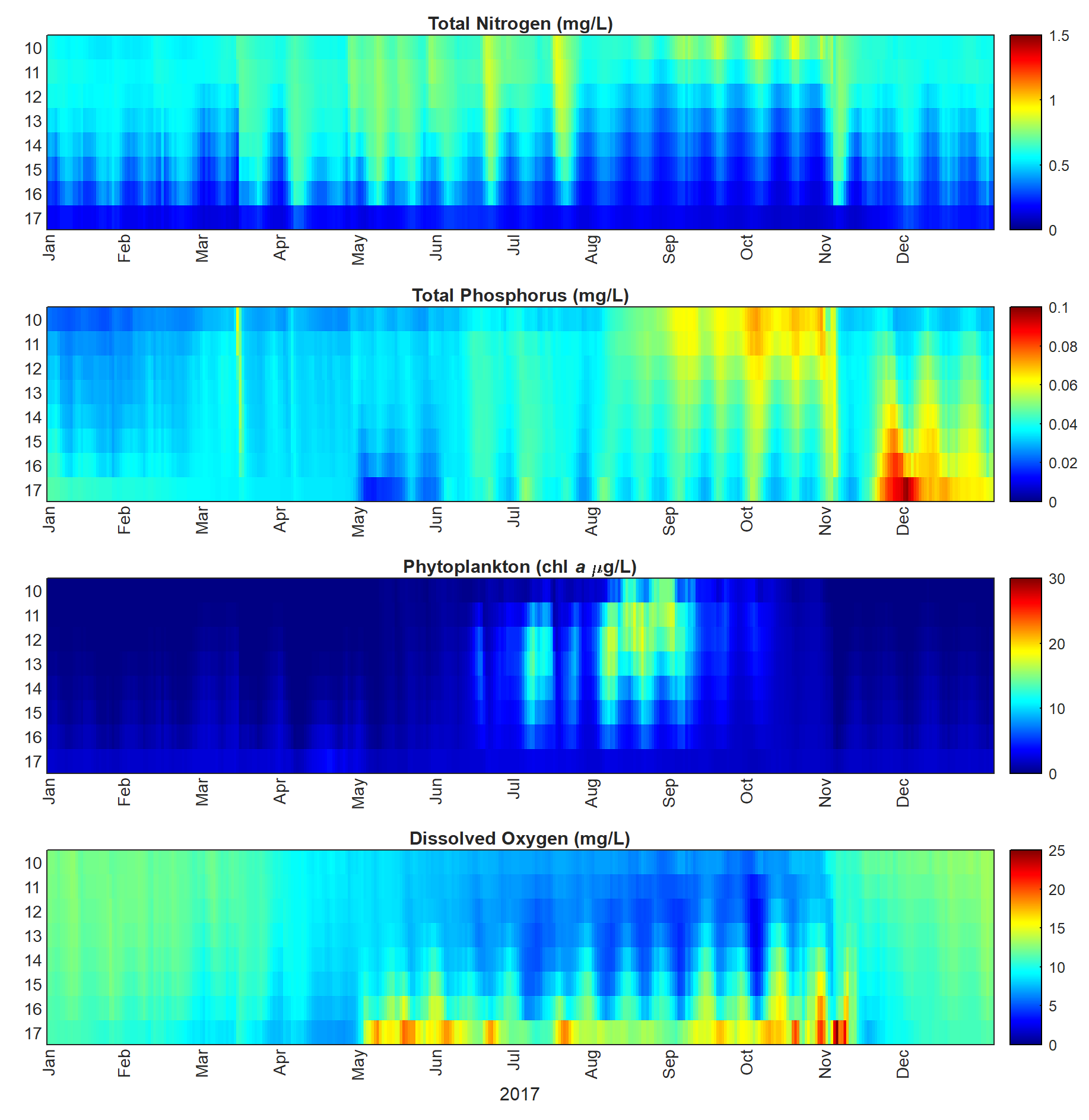


Figure S11. Heatmap of simulated concentrations of total nitrogen, total phosphorus, phytoplankton, and dissolved oxygen in the year of 2017. Data are presented for WASP segments 10-17 (y-axis).


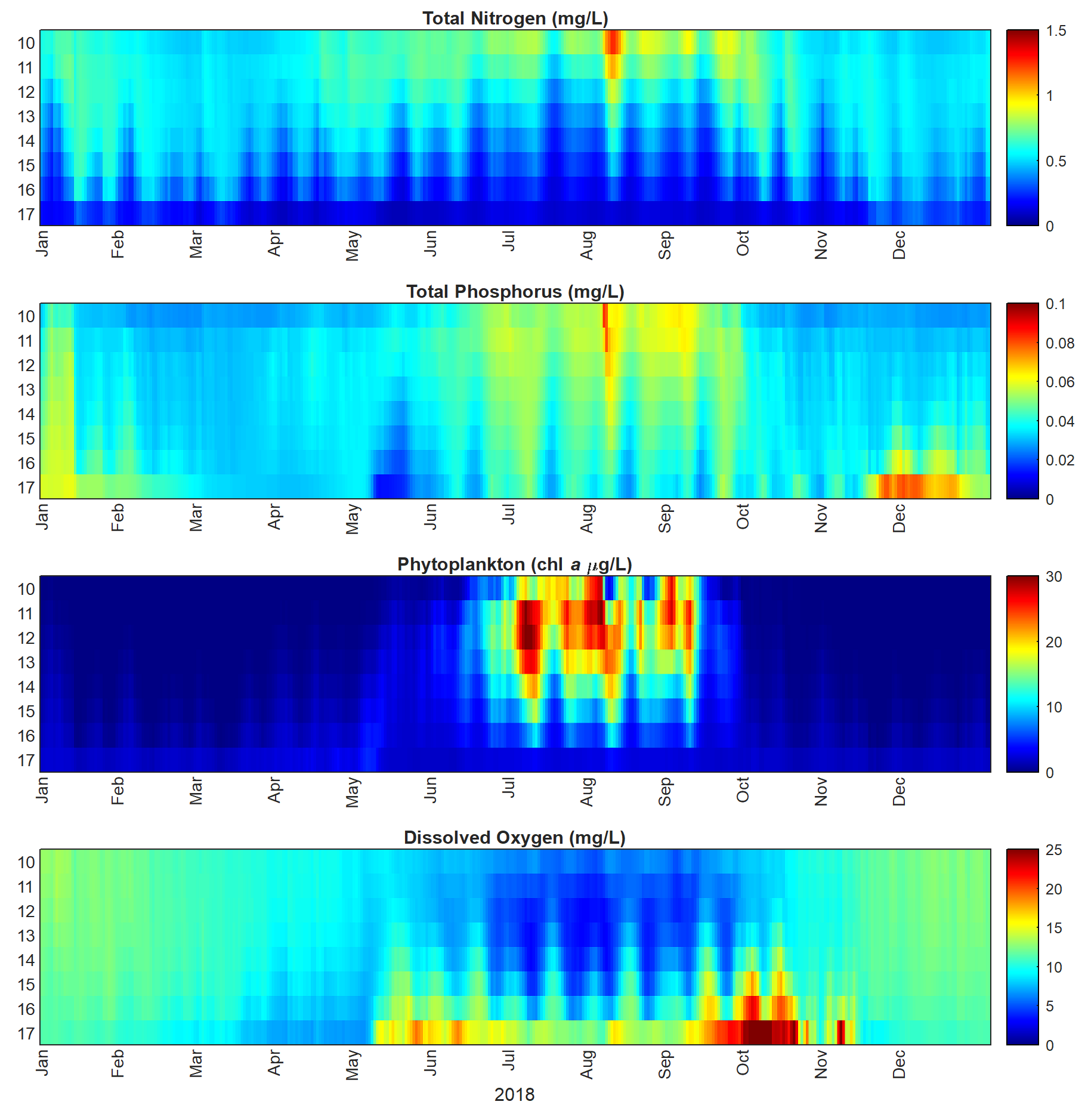


Figure S12. Heatmap of simulated concentrations of total nitrogen, total phosphorus, phytoplankton, and dissolved oxygen in the year of 2018. Data are presented for WASP segments 10-17 (y-axis).


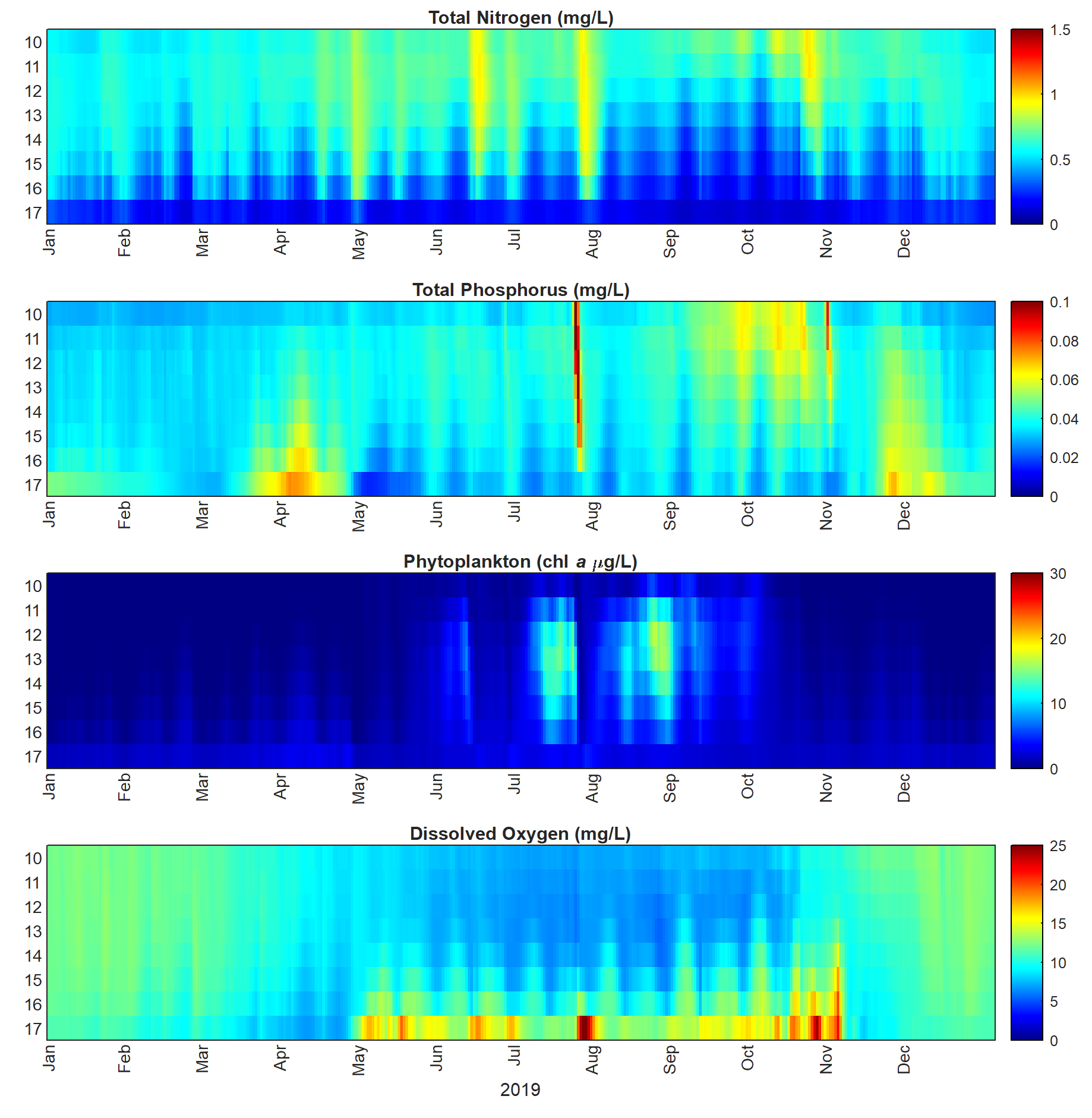


Figure S13. Heatmap of simulated concentrations of total nitrogen, total phosphorus, phytoplankton, and dissolved oxygen in the year of 2019. Data are presented for WASP segments 10-17 (y-axis).


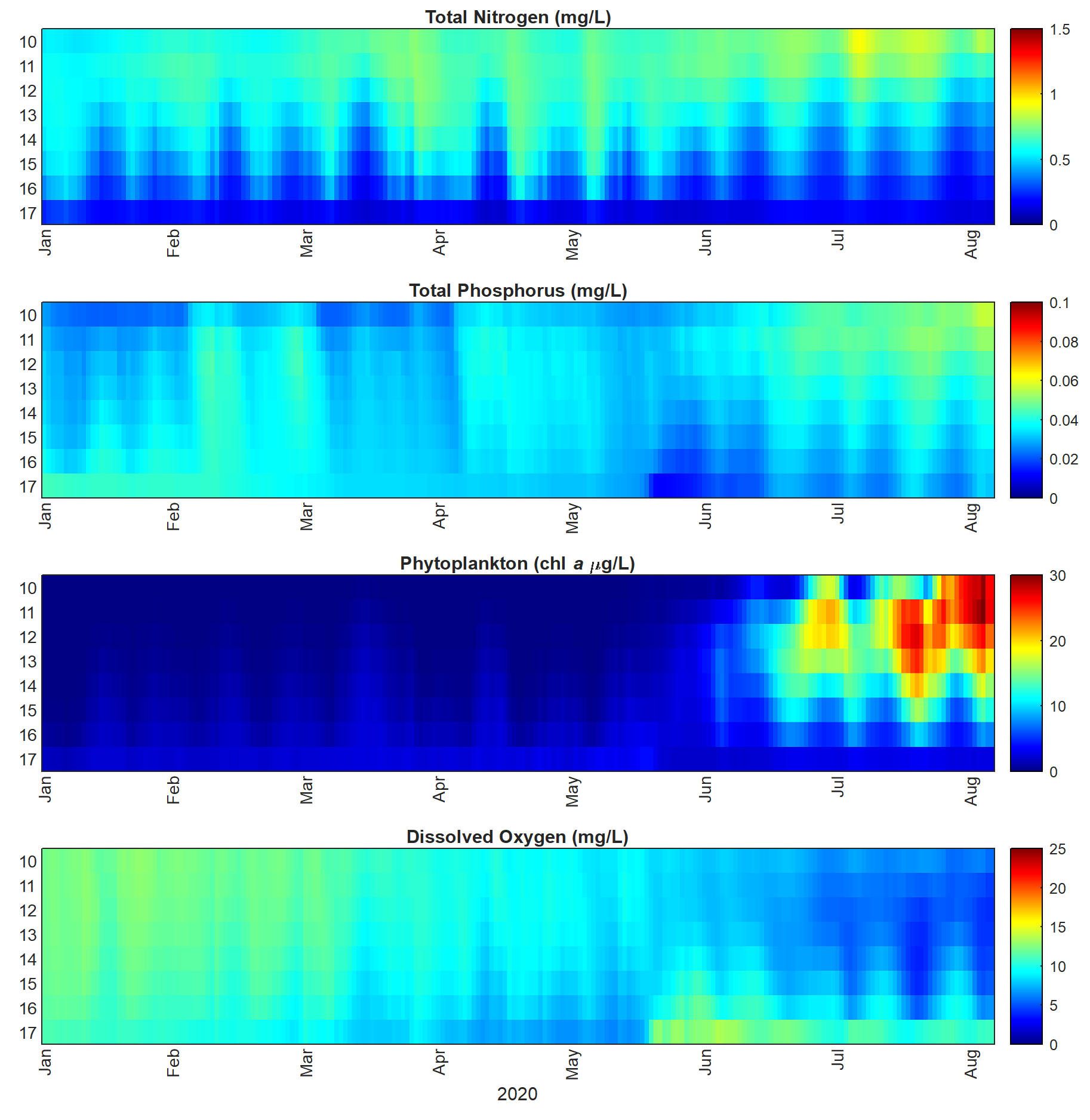


Figure S14. Heatmap of simulated concentrations of total nitrogen, total phosphorus, phytoplankton, and dissolved oxygen in the year of 2020. Data are presented for WASP segments 10-17 (y-axis).

## *5.5 Parameter Evaluation Effect on Dissolved Oxygen*


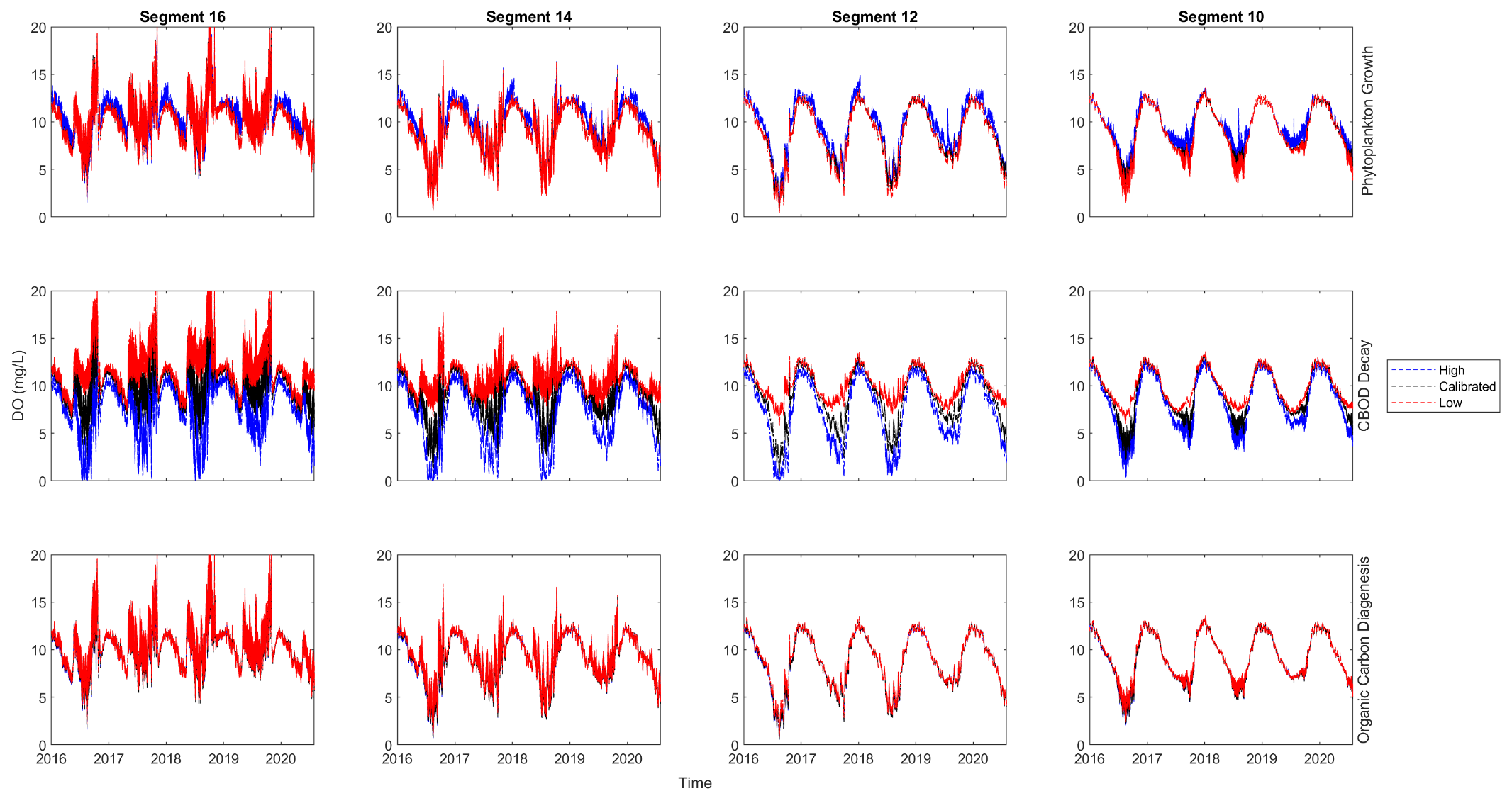


Figure S15. Sensitivity analysis of phytoplankton growth (row 1), CBOD decay (row 2), and POC diagenesis rates (row 3) on simulated DO concentrations. “High” conditions represent parameter values 10x the calibrated value and “low” conditions represent 1/10^th^ of the calibrated value. Dates presented span the entire model simulation. Locations move upstream from left to right.

We evaluated the effect on dissolved oxygen concentrations by varying three rate constants. Simulated DO for different phytoplankton growth rate constants (row 1), CBOD decay rate constants (row 2), and diagenesis of organic carbon rate constants (row 3) is presented in Figure S15. Each of these components directly affect dissolved oxygen concentrations. Calibrated rate constants were divided and multiplied by ten to create a Low (red), Calibrated (black), and High (blue) condition for each parameter. Data presented in this figure span the entire model simulation and are taken from Segments 16, 14, 12, and 10. Refer to Table S9 for the ranges and averages of [DO] presented in Figure S15.

The alteration of phytoplankton growth rate constants had a small impact on DO through the growth and respiration of larger phytoplankton blooms. The higher growth rate resulted in consistently higher average [DO]. The difference between Low and High conditions were larger at Segment 14 (0.71 mg/L) and 12 (0.88 mg/L) compared to Segment 10 (0.53 mg/L) and Segment 16 (0.2 mg/L). This illustrates that mid-stream segments are more sensitive to alterations of phytoplankton growth than those further upstream. Changes in Segment 16 are likely due to lower phytoplankton concentrations. The larger blooms increased the amount of DO being produced while the lower conditions had lower minimal values and not as much activity in the summer months.

The CBOD decay rate constant had the largest impact on [DO] in the water column and was most sensitive to changes in parameterization. The results suggest that simulated [DO] at the higher decay rate constant were consistently lower than the calibrated value, and the lower CBOD decay rate resulted in a simulated [DO] that was on average 1.71 mg/L lower than the calibrated value. Further, the differences between the High and Calibrated conditions decrease moving upstream, suggesting that the impact of CBOD is less relevant in the upstream sections of the PRE. The differences between the Low and Calibrated conditions are less than those of the High and Calibrated conditions. Additionally, the impact of these adjusted rate constants varies with each year and by the seasons. Simulations indicate that CBOD decay has a larger impact in the summer months and there is also yearly variation within each segment.

Alteration of the sediment diagenesis rate constants of particulate organic carbon (POC) had a small impact in each segment. Simulated results suggest that the High condition consistently produces a lower mean [DO], although results of the High condition are much closer to those of the Calibrated condition compared to the Low. This suggests that the calibrated rate for organic carbon diagenesis already removes most of the DO possible for this sink. Differences in SOD parameterization were more pronounced upstream of Segment 16, similarly to phytoplankton. The parameterization in this analysis did have an impact on SOD rate. The low decay rate constant of POC had max SOD rates of about 0.9 g/m^2^-day. Calibrated and high conditions were closer to 1.9-2.3 g/m^2^-day. These results, along with the observed vertical gradient of DO, suggest that SOD is likely an important contributor to hypoxia solely in the bottom waters.

Table S9. Table containing ranges and means of simulated [DO] data presented in Figure S15.

| **Parameter** | **Condition** | **Segment 16** | | **Segment 14** | | **Segment 12** | | **Segment 10** | |
| --- | --- | --- | --- | --- | --- | --- | --- | --- | --- |
|  |  | Range | Average | Range | Average | Range | Average | Range | Average |
| Phytoplankton Growth | High | 1.44-22.3 | 10.7 | 0.87-16.4 | 9.86 | 0.94-14.9 | 9.53 | 2.60-13.7 | 9.67 |
|  | Calibrated | 1.71-22.5 | 10.4 | 0.64-16.5 | 9.23 | 0.54-13.5 | 8.82 | 2.18-13.6 | 9.26 |
|  | Low | 1.86-22.4 | 10.5 | 0.58-16.5 | 9.15 | 0.45-13.5 | 8.65 | 1.32-13.6 | 9.14 |
| CBOD Decay | High | 0.02-19.9 | 7.82 | 0.02-13.4 | 7.2 | 0.02-13.2 | 7.4 | 0.34-13.4 | 8.45 |
|  | Calibrated | 1.71-22.5 | 10.4 | 0.64-16.5 | 9.23 | 0.54-13.5 | 8.82 | 2.18-13.6 | 9.26 |
|  | Low | 6.75-24.3 | 11.6 | 6.61-17.9 | 10.8 | 5.78-13.6 | 10.1 | 5.96-13.6 | 9.83 |
| Organic Carbon Diegensis | High | 1.60-22.3 | 10.4 | 0.61-16.4 | 9.2 | 0.52-13.5 | 8.8 | 2.05-13.6 | 9.25 |
|  | Calibrated | 1.71-22.5 | 10.4 | 0.64-16.5 | 9.23 | 0.54-13.5 | 8.82 | 2.18-13.6 | 9.26 |
|  | Low | 2.14-22.9 | 10.5 | 0.91-16.9 | 9.37 | 0.79-13.5 | 8.95 | 2.42-13.6 | 9.34 |

# **6. Associated Input and Observed Data**

Files listed in the below include inputs from the Wood-Pawcatuck Watershed HSPF model, atmospheric forcing functions, continuous and discrete observed data in the Pawcatuck River Estuary, and additional data used in the PRE model development. Refer to the table below for the file name and the available information. This data was made available via Mendeley.com with the associated DOI: 10.17632/vywyyks4r6.2.

| **File Name** | **Available Information** |
| --- | --- |
| 2018RIDEMSondeDataSummary PawcatuckRiver.doc | Additional information and data summary of 2018 RIDEM sonde deployments |
| 2019RIDEMSondeDataSummary PawcatuckRiver.doc | Additional information and data summary of 2019 RIDEM sonde deployments |
| 2020RIDEMSondeDataSummary PawcatuckRiver.doc | Additional information and data summary of 2020 RIDEM sonde deployments |
| DEM Pawcatuck Dock Deployment Locations.pdf | Photos of RIDEM sonde deployment location on docks |
| HSPFtoWASP_1_ReadMe_Pawc.xlsx | Descriptive file explaining HSPF input files, abbreviations, and input locations |
| HSPFtoWASP_2_Local_Pawc.xlsx | Local runoff and loads for WASP segments 1-16 |
| HSPFtoWASP_2_LocalConc_Pawc.xlsx | Outputs from previous file converted into concentrations |
| HSPFtoWASP_3_Tributary_Pawc.xlsx | Tributary flows and loads at daily timestep |
| HSPFtoWASP_3_Tributary101_Pawc.xlsx | Same output as previous file but tributaries into WASP Segment 17 are combined |
| HSPFtoWASP_3_TributaryConc_Pawc.xlsx | Calculated tributary inflow and loads as concentrations into WASP Segments 1, 5, and 17 |
| HSPFtoWASP_4_PointSources_Pawc.xlsx | Westerly and Stonington WWTP loads at daily time step |
| HSPFtoWASP_4_PointSourcesConc_Pawc.xlsx | Same output as previous file but as concentrations |
| HSPFtoWASP_5_AtmDep_Pawc.xlsx | Daily time-series for atmospheric data |
| HSPFtoWASP_6_Met_Pawc.xlsx | All meteorological data that span WASP segments at hourly timestep |
| HHSPFtoWASP_6_MetAdditional_Pawc.xlsx | Additional parameters of pressure, relative humidity, and wind direction |
| HSPFtoWASP_6_MetPrecipEvapCMS_Pawc.xlsx | Precipitation and evaporation calculated in cubic meters per second |
| LNB Grab 2019 Final.xlsx | Grab samples of nutrients, chlorophyll *a*, and water quality in various locations |
| NOAA Chart 13214.pdf | NOAA nautical chart showing LNB and Pawcatuck River depth soundings in MLLW |
| NOAA PORTS 8456620 Water Temperature Data.xlsx | Water temperature collected by NOAA 8456620 in Newport, RI |
| NOAA PORTS 8461490 Tidal Data.xlsx | Tidal height collected by NOAA 8451590 in New London, CT |
| Pawcatuck2018NutrientSampleResults.xlsx | Discrete nutrient samples and N-P ratios at various locations in Pawcatuck |
| rawdata.check.AvondaleBottom2019.xlsx | Continuous observed data, water column profile checks, and additional notes from sonde |
| rawdata.check.AvondaleBottom2020.xlsx | Continuous observed data, water column profile checks, and additional notes from sonde |
| rawdata.check.AvondaleSurface2019.xlsx | Continuous observed data, water column profile checks, and additional notes from sonde |
| rawdata.check.AvondaleSurface2020.xlsx | Continuous observed data, water column profile checks, and additional notes from sonde |
| rawdata.check.pawcatuckpoint2018.xlsx | Continuous observed data, water column profile checks, and additional notes from sonde |
| rawdata.check.Viking2018.xlsx | Continuous observed data, water column profile checks, and additional notes from sonde |
| rawdata.check.Viking2019.xlsx | Continuous observed data, water column profile checks, and additional notes from sonde |
| rawdata.check.Viking2020.xlsx | Continuous observed data, water column profile checks, and additional notes from sonde |
| rawdata.check.WYC2018.xlsx | Continuous observed data, water column profile checks, and additional notes from sonde |
| rawdata.LNBSurface2019.xlsx | Continuous observed data from sonde |
| Rawdata.PawcatuckPointSurface2019.xlsx | Continuous observed data from sonde |
| RSI-3108 Pawcatuck River Watershed HSPF Modeling Report.pdf | HSPF model development report by RESPEC including information on model background, input data, and calibration |
| SeawardBoundary_Inputs.xlsx | WASP inputs at seaward boundary acquired from various data sources |
| USGS Greenhaven Data.xlsx | Continuous observed data from sonde |
| USGS Pawcatuck Rock Data.xlsx | Continuous observed data from sonde |
| USGS Route 1 Data.xlsx | Continuous observed data from sonde |

# **7. References**

Ambrose, R.B., Wool, T.A., Martin, J.L. 1993. The Water Quality Analysis Simulation Program,

WASP5; Part A: Model Documentation. Internal Report Distributed by USEPA Center for Exposure Assessment Modeling, U.S. Environmental Protection Agency, Athens, GA.

Ambrose, R.B., Wool, T.A. 2017. WASP8 Stream Transport - Model Theory and User's Guide;

Supplement to Water Quality Analysis Simulation Program (WASP) User Documentation. U.S. EPA, Office of Research and Development: Athens, GA, USA. https://epawasp.twool.com/resources/Stream-Transport-User-Guide.pdf

Dettman, E.H., Charlestra, L., Abdelrhman, M.A. 2020. Seasonal and Diel Oxygen and

Phytoplankton Dynamics in an Estuarine Water Quality Model. United States Environmental Protection Agency. Office of Research and Development. National health and Environmental Effects Laboratory. Narragansett, RI. EPA/600/R-18/378.

Knightes, C.D., Ambrose, R.B., Avant, B., Han, Y., Acrey, B., Bouchard, D.C., Zepp, R., Wool,

T.A. 2019. Modeling framework for simulating concentrations of solute chemicals, nanoparticles, and solids in surface waters and sediments: WASP8 Advanced Toxicant Module. *Environmental Modelling & Software.* Volume 111, Pages 444-458, ISSN 1364-8152. https://doi.org/10.1016/j.envsoft.2018.10.012.

Lupo, C.D., McCutcheon, C.M., Kenner, S.J. 2022. PAWCATUCK RIVER WATERSHED

FINAL HSPF MODELING REPORT Revision 1 Topical Report RSI-3108. Prepared for Connecticut Department of Energy and Environmental Protection.

Martin, J.L., Ambrose, R.B., Wool, T.A. 2006. WASP7 Benthic Algae - Model Theory and

User's Guide. U.S. EPA, Region 4, Atlanta, GA.

Martin, J.L., Ambrose, R.B., Wool, T.A. 2018. WASP8 Macro Algae – Model Theory and

User’s Guide; Supplement to Water Quality Analysis Simulation Program (WASP) User Documentation. U.S. EPA, Office of Research and Development: Athens, GA, USA. https://epawasp.twool.com/resources/WASP-Macroalgae_manual-V3.pdf

Martin, J.L., Wool, T.A. 2017. WASP Sediment Diagenesis Routines: Model Theory and User’s

Guide. U.S. EPA, Office of Research and Development: Athens, GA, USA.

https://www.epa.gov/sites/default/files/2018-05/documents/wasp8_sod_module_v1.pdf

NOAA Office of Coast Survey. 2014. Fishers Island Sound. Harbor Chart 13214.

https://www.charts.noaa.gov/OnLineViewer/13214.shtml

Rollinson, V. R., Granger, J., Clark, S.C., Blanusa, M.L., Koerting, C.P., Vaudrey, J.M.P.,

Treibergs, L.A., Westbrook, H.C., Matassa, C.M., Hastings, M.G., Tobias, C.R. 2021. Seasonality of nitrogen sources, cycling, and loading in a New England river discerned from nitrate isotope ratios. *Biogeosciences*. Volume 18, Issue 11, Pages 3421-3444. https://doi.org/10.5194/bg-18-3421-2021

U.S. EPA. Hydrological Simulation Program – FORTRAN (HSPF). 2022. EPA Center for

Exposure Assessment Modeling. https://www.epa.gov/ceam/hydrological-simulation-program-fortran-hspf#Introduction

Vaudrey, J.M.P., Krumholz, J., Calabretta, C. 2020. DRAFT Model Report, v. 2020-11-11.

University of Connecticut, Department of Marine Sciences, Groton, CT. Prepared for the Niantic Nitrogen Work Group. 128 p.

Wool, T.A., Ambrose, R.B., Martin, J.L. 2008. WASP8 Temperature - Model Theory and User’s

Guide; Supplement to Water Quality Analysis Simulation Program (WASP) User Documentation. U.S. EPA, Office of Research and Development: Athens, GA, USA. https://www.epa.gov/sites/default/files/2018-05/documents/heat-model.pdf

Wool, T.A., Ambrose, R.B., Martin, J.L. 2011. WASP8 Multiple Algae - Model Theory and

User’s Guide; Supplement to Water Quality Analysis Simulation Program (WASP) User Documentation. U.S. EPA, Office of Research and Development: Athens, GA, USA. https://www.epa.gov/sites/default/files/2018-05/documents/mpm-user-guide.pdf

Wool, T.A., Ambrose, R.B., Martin, J.L., Comer, A. 2020. WASP 8: The Next Generation in the

50-year Evolution of US EPA’s Water Quality Model. *Water*. Volume 12, Issue 5. https://doi.org/10/3390/w12051398.

Wool, T.A. 2017. WASP8 Light Module – Model Theory and User’s Guide; Supplement to

Water Quality Analysis Simulation Program (WASP) User Documentation. U.S. EPA, Office of Research and Development: Athens, GA, USA. https://www.epa.gov/sites/default/files/2018-05/documents/light-module.pdf
